# Delineation of Diverse Macrophage Activation Programs in Response to Intracellular Parasites and Cytokines

**DOI:** 10.1371/journal.pntd.0000648

**Published:** 2010-03-30

**Authors:** Shuyi Zhang, Charles C. Kim, Sajeev Batra, James H. McKerrow, P'ng Loke

**Affiliations:** 1 Department of Pathology, University of California, San Francisco, California, United States of America; 2 Sandler Center for Basic Research in Parasitic Diseases, University of California, San Francisco, California, United States of America; 3 Department of Biochemistry and Biophysics, University of California, San Francisco, California, United States of America; 4 Department of Medical Parasitology, New York University Langone Medical Center, New York, New York, United States of America; University of Edinburgh, United Kingdom

## Abstract

**Background:**

The ability to reside and proliferate in macrophages is characteristic of several infectious agents that are of major importance to public health, including the intracellular parasites *Trypanosoma cruzi* (the etiological agent of Chagas disease) and *Leishmania* species (etiological agents of Kala-Azar and cutaneous leishmaniasis). Although recent studies have elucidated some of the ways macrophages respond to these pathogens, the relationships between activation programs elicited by these pathogens and the macrophage activation programs elicited by bacterial pathogens and cytokines have not been delineated.

**Methodology/Principal Findings:**

To provide a global perspective on the relationships between macrophage activation programs and to understand how certain pathogens circumvent them, we used transcriptional profiling by genome-wide microarray analysis to compare the responses of mouse macrophages following exposure to the intracellular parasites *T. cruzi* and *Leishmania mexicana*, the bacterial product lipopolysaccharide (LPS), and the cytokines IFNG, TNF, IFNB, IL-4, IL-10, and IL-17. We found that LPS induced a classical activation state that resembled macrophage stimulation by the Th1 cytokines IFNG and TNF. However, infection by the protozoan pathogen *L. mexicana* produced so few transcriptional changes that the infected macrophages were almost indistinguishable from uninfected cells. *T. cruzi* activated macrophages produced a transcriptional signature characterized by the induction of interferon-stimulated genes by 24 h post-infection. Despite this delayed IFN response by *T. cruzi*, the transcriptional response of macrophages infected by the kinetoplastid pathogens more closely resembled the transcriptional response of macrophages stimulated by the cytokines IL-4, IL-10, and IL-17 than macrophages stimulated by Th1 cytokines.

**Conclusions/Significance:**

This study provides global gene expression data for a diverse set of biologically significant pathogens and cytokines and identifies the relationships between macrophage activation states induced by these stimuli. By comparing macrophage activation programs to pathogens and cytokines under identical experimental conditions, we provide new insights into how macrophage responses to kinetoplastids correlate with the overall range of macrophage activation states.

## Author Summary

Macrophages are a type of immune cell that engulf and digest microorganisms. Despite their role in protecting the host from infection, many pathogens have developed ways to hijack the macrophage and use the cell for their own survival and proliferation. This includes the parasites *Trypanosoma cruzi* and *Leishmania mexicana*. In order to gain further understanding of how these pathogens interact with the host macrophage, we compared macrophages that have been infected with these parasites to macrophages that have been stimulated in a number of different ways. Macrophages can be activated by a wide variety of stimuli, including common motifs found on pathogens (known as pathogen associated molecular patterns or PAMPs) and cytokines secreted by other immune cells. In this study, we have delineated the relationships between the macrophage activation programs elicited by a number of cytokines and PAMPs. Furthermore, we have placed the macrophage responses to *T. cruzi* and *L. mexicana* into the context of these activation programs, providing a better understanding of the interactions between these pathogens and macrophages.

## Introduction

Macrophages are innate immune cells that respond to a variety of stimuli [1], [2]. In the early, acute phase of an infection, they are activated by pathogen-associated molecular patterns (PAMPs), allowing them to recognize, engulf, and kill invading pathogens [3]. During the chronic phase of infection, macrophages are further activated by cytokines secreted by T cells [4]. Interaction with different PAMPs and cytokines leads to different states of macrophage activation [5]. These include innate macrophage activation by microbial products such as LPS through engagement of pattern recognition receptors (PRRs), classical macrophage activation by T helper 1 (Th1) cytokines such as interferon gamma (IFNG) and tumor necrosis factor (TNF), alternative macrophage activation by T helper 2 (Th2) cytokines such as interleukin-4 (IL-4) and interleukin-13 (IL-13), and macrophage “deactivation” by interleukin-10 (IL-10) [6], tumor growth factor beta (TGF-B) or phagocytosis of apoptotic cells [7] and Fc-receptor (FcR) crosslinking [1],[5],[8].

Although many different states of macrophage activation (or deactivation) have been identified, the phenotypic relationships between these states remain unclear at a molecular level. Previous studies have used transcriptional profiling to determine gene expression in macrophages after they are activated with bacteria [9],[10], type I/II interferons [11], and various intracellular parasites [12],[13],[14],[15],[16],[17],[18],[19],[20],[21],[22]. However, because all of these experiments were performed separately, they cannot be easily compared and do not directly address the phenotypic relationship between the different states of macrophage activation. To clearly determine how the different states of macrophage activation relate to one another under otherwise identical culture conditions, we compared the transcriptional response of bone marrow-derived macrophages to infection by the kinetoplastid intracellular parasites *Leishmania mexicana* and *Trypanosoma cruzi*, stimulation by the bacterial PAMP lipopolysaccharide (LPS), and stimulation by the cytokines IFNG, TNF, IFNB, IL-4, IL-10, and IL-17. Additionally, in order to determine whether different types of macrophages respond differently to activation stimuli, we compared the transcriptional responses of thioglycollate-elicited peritoneal macrophages with transcriptional responses of identically treated bone-marrow derived macrophages following stimulation with IFNG, IL-4, and TNF.

## Methods

### Macrophage preparation

Bone marrow-derived macrophages (BMMs) were differentiated from marrow isolated from femurs and tibias of five 6–8 week-old C57BL/6 mice (Charles River). The cells were pooled and cultured in BMM media composed of DMEM + 20% FBS + 10% 3T3 supernatant containing MCSF + 1% Na Pyruvate + 1% L-glutamine + 1% Penicillin-streptomycin (BMM media). The cells were differentiated for 6 days, harvested and frozen down in 90% FBS + 10% DMSO. Cryopreserved macrophages were used so that all experiments could be conducted using the same batch of cells. The transcriptional signature of cryopreserved macrophages was compared to that of fresh macrophages to ensure the quality of the frozen and thawed BMMs used in these experiments (Figure S1). The purity of BMMs was confirmed by flow cytometry analysis using lymphocyte, granulocyte, monocyte, and dendritic cell surface markers (Figure S2). Replicate experiments were performed using a separate batch of BMMs derived from a different set of five C57BL/6 mice. One day before infection, macrophages were thawed and plated on T25 flasks at a density of 5×10^6^ cells per flask in BMM media.

Thioglycollate-elicited macrophages were derived by intraperitoneal injection of five C57BL/6 mice with 2.5 mL sterile thioglycollate. Mice were sacrificed 72 h post-injection. Peritoneal lavage was performed by washing the cavity twice with 5 mL of PBS. Cells were washed and plated in DMEM + 10% FBS. Experiments using thioglycollate-elicited macrophages were performed the following day after removal of non-adherent cells by repeated washing with PBS.

### Macrophage infections and stimulation

*Leishmania mexicana* (strain MNYC/BZ/62/M379) were grown in M199 media and were washed and resuspended in DMEM + 0.5% FBS for infection. *Trypanosoma cruzi* (strain CAI-72) were seeded on a monolayer of BESM (Bovine Embryo Skeletal Muscle) cells (grown in RPMI + 20% FBS). On day 5 after seeding, the media on the cells was replaced, and metacyclic trypomastigotes were collected the following day. *T. cruzi* parasites were then washed and resuspended in DMEM + 0.5% FBS for infection. For *L. mexicana* and *T. cruzi* infections, BMMs were washed once with D-PBS, and their media was replaced with DMEM + 0.5% FBS containing parasites at a MOI of 10 (an MOI of 1 resulted in only 10% of BMMs infected, and an MOI of 50 resulted in extensive lysis by 24 h post-infection). Control, uninfected cells received media without parasites. The flasks were centrifuged at 168xG for 5 m to synchronize the infection. All infections took place over a 24 h time course with RNA collection at 2 h, 6 h, 12 h, and 24 h time points post-infection. Our data represents three biological replicates of *L. mexicana* infection and two biological replicates of *T. cruzi* infection. Each biological replicate was performed independently using macrophages derived from a different group of mice.

Macrophages were stimulated with 100 ng/mL LPS (Sigma), 100 ng/mL IFNG (R&D Systems), 20 ng/mL IL-4 (Peprotech), 10 ng/mL of IL-10 (Peprotech), 10 ng/mL TNF (R&D Systems), 100 units/mL IFNB (Fischer Scientific), or 100 ng/mL IL-17 (Peprotech). RNA was collected at 2 h, 6 h, 12 h, and 24 h time points.

### Microarray analysis

BMMs were lysed using the TRIzol Reagent (Invitrogen), and RNA was isolated using the RNeasy Mini Kit (Qiagen). RNA was then amplified using the Amino Allyl MessageAmp II aRNA Amplification Kit (Ambion).

All microarray analysis was performed on custom printed Mouse Exonic Evidence-based Oligonucleotide (MEEBO) Arrays. Amplified RNA from each sample was hybridized against a pooled reference consisting of an equal quantity of RNA from all of the time points within a particular infection time course. The arrays were scanned using a GenePix 4000B scanner and GenePix PRO version 4.1 (Axon Instruments/Molecular Devices). The Spotreader program (Niles Scientific) was used for array gridding and image analysis. The resulting data files were uploaded to Acuity version 4.0 (Molecular Devices), where the raw data was log transformed, filtered for “good quality spots” (((‘RgnR∧2(635/532)’>0.6) AND (‘Flags’> = 0))AND((‘F532Mean-b532’>200) OR (‘F635Median-b635’>200))), normalized to the 0 h control, and filtered for data present in at least 70% of samples. The resulting dataset (Dataset S1) was then analyzed for statistically significant genes using the Statistical Analysis of Microarray (SAM) software version 3.0 (available at http://www-stat.stanford.edu/~tibs/SAM/).

Microarrays that were of poor quality (high background, low foreground) were repeated.

### Statistical analysis of microarray data

Pairwise comparisons were performed between infected/stimulated cells and uninfected cells in order to determine the number of genes significantly affected by the infection/stimulation and the relative fold changes of these genes. To do this, the two-class unpaired analysis in SAM was employed with a false discovery rate (FDR) cutoff of 1% and the condition that genes must have at least a two-fold change. This stringent FDR cutoff was chosen in order to focus on genes most highly induced or repressed by each condition. We treated each time point as an independent replicate to identify genes that were consistently up or down-regulated over the 24 h time course. Data for *L. mexicana*-infected, *T. cruzi*-infected, and uninfected BMMs included 3, 2, and 5 replicate time courses, respectively. Biological replicates of *L. mexicana*-infected, *T. cruzi*-infected, or uninfected cells were treated as replicates for the purposes of this analysis.

Multiclass comparisons were also performed between infected/stimulated cells to determine the number of genes significantly different between these groups by multiclass analysis in SAM with a false discovery cutoff of 0.1%. Biological replicates of *L. mexicana* and *T. cruzi* were treated as replicates for the purposes of this analysis. Once a list of significant genes was obtained, data was extracted from the total dataset for this list of significant genes using the Samster tool [23]. Data from each of the biological replicates were averaged for each individual time point, filtered for 90% present data, and hierarchically clustered in Cluster version 3.0 (http://bonsai.ims.u-tokyo.ac.jp/~mdehoon/software/cluster/software.htm#ctv). The resulting heat map and trees were visualized using Java Treeview (available at http://sourceforge.net/projects/jtreeview/files/).

Gene ontology analysis was performed using the PANTHER (Protein Analysis Through Evolutionary Relationships) classification system (available at http://www.pantherdb.org/). Genes upregulated by one or multiple stimuli were input into PANTHER along with a background gene list representing all of the genes used in the SAM analysis. The biological processes that were over-represented in the genes of interest were identified and sorted based on classification type and *p*-value.

### *Leishmania* and *Trypanosoma* meta-analysis

For studies that have original array files available in a public database, those files were obtained and used for processing. For studies that did not have original array files available, we relied on the authors' preprocessed data.

Data were log_2_ transformed if necessary and arrays were median centered. Biological and technical replicates, if available, were averaged for each time point for infected samples. All infected samples for each probe expression value were then subtracted by either their paired uninfected sample or the time zero expression value.

For data generated by this study, each array was median centered and biological replicates for each time point for both infected and uninfected were averaged. The corresponding uninfected expression values were subtracted from infected.

All the columns in the integrated data sets were Z-score transformed and 90% present filtered. SAM one class analysis was performed with a 1% FDR cutoff. HUGO identifiers were converted to Entrez identifiers for functional analysis in PANTHER. Methods for supplemental materials have been provided as Methods S1.

### Accession numbers

Microarray data have been deposited in GEO under accession number GSE20087.

## Results

### Muted macrophage activation signature by kinetoplastids relative to LPS

Macrophages respond to pathogens through engagement of pattern recognition receptors (PRRs), the most well characterized being the Toll-like receptors (TLRs) [3],[24]. Innate activation through engagement of TLR4 by LPS [25] is well characterized as being responsible for the majority of the activation program induced by gram-negative bacteria [9]. However, intracellular protozoan pathogens induce macrophage responses that are distinct from their bacterial counterparts [19]. In order to compare innate macrophage activation programs, bone marrow-derived macrophages (BMM) were infected with the intracellular protozoan pathogens, *Leishmania mexicana* and *Trypanosoma cruzi*, or stimulated with LPS, and host expression responses were analyzed using microarrays.

**Figure 1:**
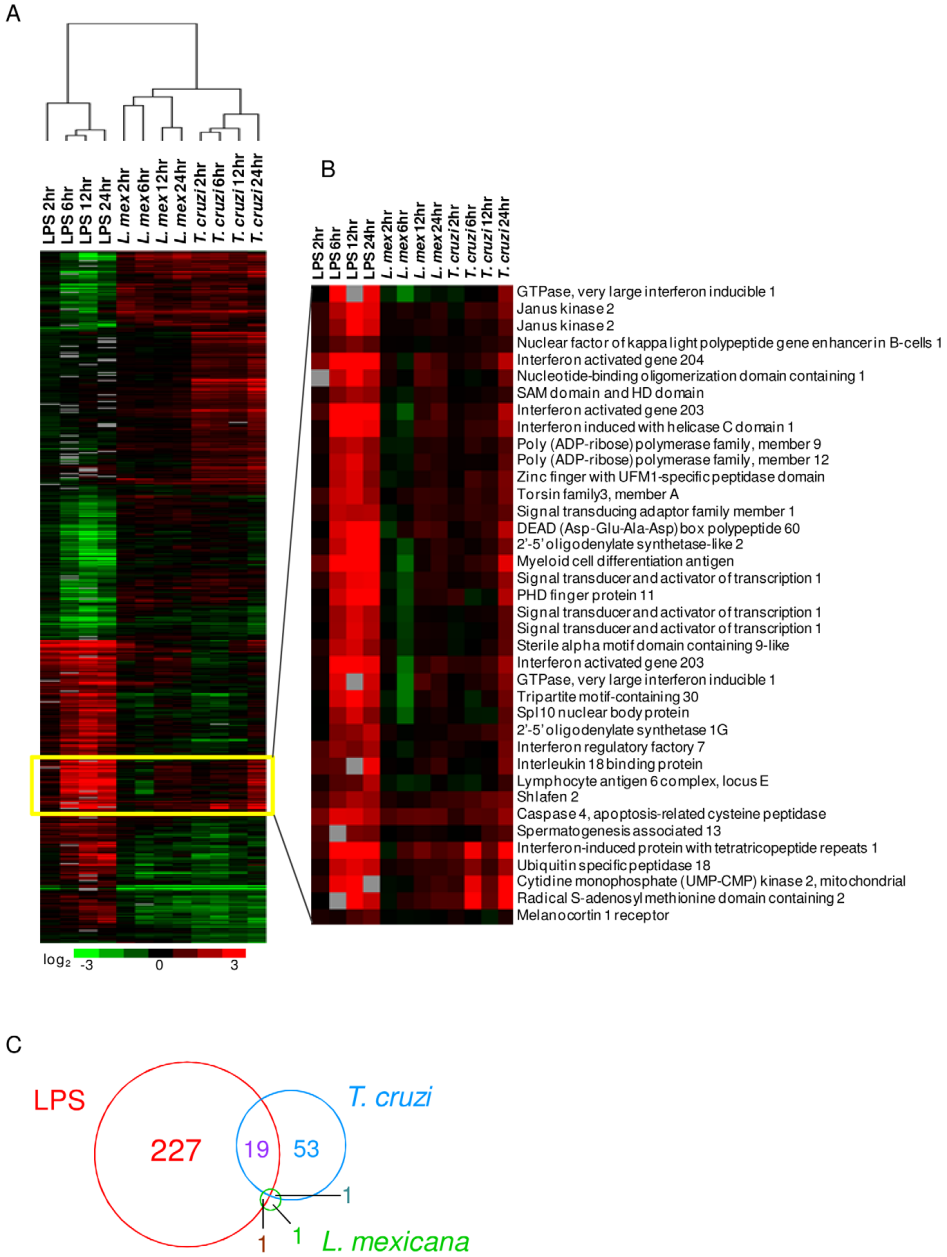
Comparison of transcriptional responses following infection by the intracellular pathogens *Leishmania mexicana* and *Trypanosoma cruzi* and following stimulation by LPS. A. Unsupervised two-dimensional cluster analysis was performed on genes exhibiting statistically significant variability between the three conditions, as determined by multiclass SAM (n = 636). Replicate experiments of *L. mexicana* (n = 3) and *T. cruzi* (n = 2) infection were averaged prior to cluster analysis. B. Close-up of gene cluster upregulated by LPS and the 24 h timepoint of *T. cruzi*. This cluster includes many interferon-stimulated genes which are not induced by *L. mexicana*. C. The Venn diagram depicts the overlap of genes significantly upregulated, as determined by pairwise SAM analysis to uninfected controls, by *L. mexicana*, *T. cruzi*, LPS, both *T. cruzi* and LPS, both *T. cruzi* and *L. mexicana*, and both *L. mexicana* and LPS. There were no genes significantly upregulated by all three conditions.

In comparison to uninfected control cells, *L. mexicana* infection of BMMs resulted in few changes in gene expression (Figure 1B), which is consistent with other reports describing the subtle nature of *Leishmania* infection [13],[16],[18],[19],[20],[26]. . This lack of response by the infected macrophages was not due to the absence of infectivity by the parasites, as both flow cytometry and microscopy revealed that BMMs were effectively infected by *L. mexicana* (**F**igure S3 A–E).

*T. cruzi* differed from *L. mexicana* in that it induced a number of genes by 24 h post-infection, many of which are known interferon-stimulated genes (Figure 1A). These results were confirmed by quantitative real-time PCR (qPCR) analysis for two interferon-stimulated genes including interferon-induced protein with tetratricopeptide repeats 3 (*Ifit3*) (Figure S4 A) and interferon activated gene 205 (*Ifi205*) (Figure S4 B). This late activation of an IFN response may correlate with *T. cruzi* escape from sequestration in a parasitophorous vacuole into the host cell cytosol and is consistent with previous microarray studies on *T. cruzi* infection [17],[21],[22].

In order to ensure that the transcriptional response to *T. cruzi* infection was not being affected by the parasites having been cultured in BESM cells, we compared the transcriptional signature of BMMs treated with supernatant from uninfected BESM cells (mock-infected BMMs) to the uninfected BMMs used in our experiments. The transcriptional signature of mock-infected BMMs was highly correlated with the transcriptional signature of uninfected BMMs (Figure S5).

The transcriptional response to LPS stimulation was distinct from the responses to either of the intracellular parasites (Figure 1A and 1B). Out of the 247 genes significantly induced by LPS, only 19 were also induced by *T. cruzi*, and 1 was also induced by *L. mexicana*. Genes induced by both LPS and *T. cruzi* include *Ifit1*, *Ifit2*, *Ifit3*, *Ifi204*, *Ifi44*, *Isg15*, *Isg20*, *Gbp3*, and *Gbp6* (Table 1). The induction of these interferon response genes is consistent with studies showing that *T. cruzi* infection can lead to the induction of IFNB via signalling through host PRRs [27],[28]. However, this IFN response is quite restricted relative to the response to LPS. As expected, gene ontology analysis showed that LPS induced genes enriched for a number of biological processes related to the immune response such as immunity and defense, interferon-mediated immunity, cytokine and chemokine mediated signalling pathway, macrophage-mediated immunity, T-cell mediated immunity, JAK-STAT cascade, and granulocyte-mediated immunity (Table 2). However, genes upregulated by the protozoan pathogens alone were not significantly enriched for any known biological processes.

**Table 1:** Comparison of genes induced by *L. mexicana*, *T. cruzi*, and LPS.

Pathogen/pathogen product	Expression ratio (log2) of genes induced by one pathogen/pathogen product only		
	>3	2–3	1–2
*L. mexicana*			*Mt2*
*T. cruzi*	*Ccdc54, Derl1, Lce1i*	*Col16a1, Sycn, Wipi1*	*0610010O12Rik, 1700029M20Rik, 1700049J03Rik, 1700113H21Rik, 2300005B03Rik, 2610007O09Rik, 2610034N15Rik, 2810030D12Rik, 9430076C15Rik, A130038J17Rik, Angptl4, Apba2, Atp1b1, BC023814, Bcl6b, Bhlhe41, Cbfa2t3, Chrna4, Dio2, EG330070, Ebag9, Fibcd1, Hspb8, Ifrd1, Igdcc3, Inpp4a, Mcts2, Naca, Nat15, Nat6, Olfr380, Osm, Ppm2c, Ppp1r1c, Prss33, Rgs1, Rnf216, Slc25a19, Slc25a22, Snx5, Sphk1, Tbl3, Tnfrsf9, Tspan17, Ugt1a10, Usp42, Zbtb48*
LPS	*Ccl12, Ch25h, Cxcl1, Nfkbiz*	*Adora2b, Bcl2a1c, Cav1, Ccl4, Ctsc, Cxcl2, Ddx60, Dusp1, Ell2, Ets2, Fcgr1, Fos, Glrx, Gpr85, Gvin1, Hspa1b, Ifi205, Ifih1, Jag1, Lcp2, Lpar1, Marcksl1, Ms4a6b, Oasl2, Plek, Slco3a1, Slfn4, Slfn5, Socs3, Stat1, Trim30*	*1200003I10Rik, 1200016E24Rik, 9230105E10Rik, AI451617, Abca1, Adar, Agrn, Aif1, Ak3l1, Alas1, Arhgef3, Ass1, Axud1, Azi2, Bcl2a1b, Bst1, C330023M02Rik, Casp4, Ccdc50, Ccr5, Cd302, Cd40, Cd52, Cd69, Cdkn1a, Chst7, Clec4e, Clec5a, Clic4, Cpd, Creb5, Ctla2b, Cx3cr1, Cxcl16, Cybb, Cysltr1, D14Ertd668e, Dck, Dcp2, Ddx58, Dtx3l, Dusp16, Ehd1, Eif2ak2, Epsti1, Errfi1, Fas, Fbxw17, Fgr, Filip1l, Flrt3, Fmnl2, Fndc3a, Gbp2, Gbp5, Gch1, Gda, Ggct, Glipr2, Gpd2, Gpr84, H2-T9, Herc5, Hivep3, Hmgn3, Hspa5, Ier3, Ifi203, Ifi35, Ifi47, Ift57, Igtp, Ikbke, Il15, Il15ra, Il18, Il18bp, Il1rn, Irak3, Irf1, Irf7, Irf9, Irgm1, Itga5, Jak2, Jdp2, Kctd12, Klf7, Lgals9, Lmo4, Lpcat2, Lrrc8c, Ly6a, Ly6e, Magohb, Marco, Mfsd7a, Mitd1, Mlkl, Mmp13, Mmp14, Msr1, Mx1, Mx2, N4bp1, Nfkbia, Nfkbie, Nmi, Nod1, Nod2, Oas1g, Oas2, Oasl1, Parp12, Parp14, Parp9, Pcdh7, Pde4b, Phf11, Phlda1, Pid1, Pilra, Pilrb1, Pion, Pla2g16, Pla2g4a, Plaur, Pml, Pnpt1, Pnrc1, Pols, Ppap2a, Psmb9, Psme1, Psme2, Pstpip2, Rap2c, Rapgef2, Rasgef1b, Rbpj, Rel, Ripk2, Rnf114, Rnf213, Samd9l, Samhd1, Samsn1, Sdc3, Sdc4, Sgk3, Skil, Sla, Slamf9, Slc15a3, Slc28a2, Slc2a1, Slc2a6, Slc31a2, Slpi, Smpdl3b, Sp100, Sp110, Spred1, St7, Stap1, Stat2, Stx11, Tank, Tgfbi, Tlr1, Tmem2, Tmem49, Tnf, Tnfaip3, Tnfsf9, Tor3a, Tpm4, Traf1, Trex1, Trim13, Trim21, Trim34, Ttc39c, Ube2l6, Usp25, Vasp, Zc3h11a, Zc3h12c, Zfp263, Zfp36, Zfp800, Zufsp*
**Specific genes induced by more than one pathogen/pathogen product (expression ratio varies)**			
*L. mexicana* and *T. cruzi*		*4930578M01Rik*	
*L. mexicana* and LPS		*Ccl7*	
*T. cruzi* and LPS		*Ccl2, Cmpk2, Csf1, Cxcl10, Dhx58, Gbp3, Gbp6, I830012O16Rik, Ifi204, Ifi44, Ifit1, Ifit2, Ifit3, Irgm2, Isg15, Isg20, Mnda, Rsad2, Usp18*	

**Table 2:** Comparison of biological processes induced by *L. mexicana*, *T. cruzi*, and LPS.

Biological processes induced by one or more pathogen/pathogen product			
	P value	Biological process	Genes
**LPS only**	1.65E-19	Immunity and defense	*Adora2b, Aif1, Ccl12, Ccl4, Ccr5, Cd40, Cd69, Clec4e, Clec5a, Cx3cr1, Cxcl1, Cysltr1, Dusp16, Fcgr1, Fgr, Gbp2, Gbp5, H2-T9, Hspa5, Ier3, Ifi203, Ifi205, Ifi35, Ikbke, Il15, Il15ra, Il18, Il1rn, Irak3, Irf1, Irf7, Isgf3g, Jag1, Lgals9, Lrrc8c, Marco, Msr1, Nfkbia, Nmi, Nod1, Nod2, Oas1g, Oas2, Oasl1, Oasl2, Pla2g4a, Plaur, Rel, Samhd1, Sdc4, Sla, Slamf9, Slfn5, Stat2, Tnf*
	4.41E-13	Interferon-mediated immunity	*Gbp2, Gbp5, Ifi203, Ifi205, Ifi35, Irf1, Isgf3g, Nmi, Oas1g, Oas2, Oasl1, Oasl2, Stat2*
	4.63E-06	Cytokine and chemokine mediated signaling pathway	*Ccl12, Ccl4, Ccr5, Cd40, Cx3cr1, Cxcl1, Cxcl2, Fas, Il15, Il5ra, Il18, Il1rn, Tlr1, Tnf, Traf1*
	2.82E-05	Macrophage-mediated immunity	*Adora2b, Clec42, Cxcl1, Cxcl16, Cxcl2, Fcgr1, Gbp2, Gbp5, Msr1, Sdc4, Stat2*
	3.45E-05	Apoptosis	*Adora2b, Arhgef3, Axud1, Bcl2a1c, Casp4, Ddx58, Fas, Ifih1, Ift57, Il15, Jak2, Lgals9, Nfkbia, Nod1, Nod2, Rel, Ripk2, Sgk3, Socs3, Tnf, Traf1*
	1.03E-04	Signal transduction	*Adora2b, Agrin, AI586015, Arhgef3, Azi2, Cav1, Ccl12, Ccl4, Ccr5, Cd40, Creb5, Cx3cr1, Cxcl1, Cxcl2, Cysltr1, Dusp1, Edg2, Errfi1, Ets2, Fas, Fcgr1, Fgr, Flrt3, Gpr84, Gpr85, Hrasls3, Ifi35, Ikbke, Il15, Il15ra, Il18, Il1rn, Irak3, Jak2, Lcp2, Marcksl1, Marco, Nfkbia, Nmi, Pcdh7, Pde4b, Ppap2a, Pstpip2, Rap2c, Rapgef2, Rasgef1b, Rel, Ripk2, Sgk3, Sla, Socs3, Stat2, Tgfbi,Tlr1, Tnf, Traf1, Zfp36*
	5.73E-04	T-cell mediated immunity	*Adora2b, Dc40, Cd69, Cxcl1, Cxcl2, H2-T9, Ifi35, Il15ra, Nmi, Sla, Slamf9, Slfn5*
	1.54E-03	Intracellular signaling cascade	*AI586015, Izi2, Creb5, Cxcl1, Cxcl2, Dusp1, Dusp16, Edg2, Fgr, Ifi35, Il15, Il15ra, Il18, Jak2, Lcp2, Marcksl1, Nfkbia, Nmi, Pstpip2, Rap2c, Rapgef2, Rel Ripk2, Sgk3, Socs3, Stat2, Zfp36*
	3.11E-03	JAK-STAT cascade	*Ifi35, Il15, Il15ra, Il18, Jak2, Nmi, Socs3, Stat2*
	3.87E-03	NF-kappaB cascade	*Azi2, Cxcl1, Cxcl2, Ikbke, Nfkbia, Real, Ripk2*
	6.57E-03	Ligand-mediated signaling	*Adora2b, Ccl12, Ccl4, Cxcl1, Cxcl2, Edg2, Il15, Il15ra, Il18, Il1rn, Marco, Tnf*
	9.24E-03	Cell proliferation and differentiation	*AI586015, Aif1, Cdkn1a, Cxcl1, Cxcl2, Errfi1, Ets2, Fgr, Ifi203, Ifi205, Jag1, Jak2, Nfkbia, Pcdh7, Pa2g4a, Rel, Sdc3, Slfn5, Tgfbi, Trim13*
	1.61E-02	Granulocyte-mediated immunity	*Ccr5, Cd69, Cx3cr1, Cxcl1, Cxcl2*
	4.37E-02	Cell surface receptor mediated signal transduction	*Adora2b, AI586015, Cav1, Ccl12, Ccl4, Ccr5, Cd40, Cx3cr1, Cxcl1, Cxcl2, Cysltr1, Edg2, Fas, Flrt3, Gpr84, Gpr85, Il15, Il15ra, Il18, Il1rn, Irak3, Jak2, Ppap2a, Rapgef2, Rasgef1b, Sla, Tlr1, Tnf, Traf1*
	4.66E-02	Induction of apoptosis	*Fas, Ift57, Lgals9, Nod1, Nod2, Ripk2, Tnf, Traf1*
***T. cruzi*** ** and LPS**	5.73E-11	Interferon-mediated immunity	*Cxcl10, Gbp3, Gbp6, Ifit1, Ifit2, Ifit3*
	9.38E-06	Immunity and defense	*Ccl2, Csf1, Cxcl10, Gbp3, Gbp6, Ifit1, Ifit2, Ifit3*
	4.84E-04	Macrophage-mediated immunity	*Csf1, Cxcl10, Gbp3, Gbp6*

These results indicate that activation by LPS is far more robust than activation by these intracellular kinetoplastids. *L. mexicana* infection of macrophages in particular appears to be transcriptionally “silent,” suggesting either that the parasite lacks PAMPs, or that the parasite can inhibit either cellular signalling or host transcription. Although *T. cruzi* induced several immune-related IFN response genes, many of the genes significantly induced by *T. cruzi* infection are unnamed (having only a RIKEN designation) and have unknown functions (Table 1). These results illustrate our poor understanding of kinetoplastid macrophage interactions relative to TLR signalling in response to LPS.

### Meta-analysis of transcriptional responses to *L. mexicana* and *T. cruzi*

**Figure 2 pntd-0000648-g002:**
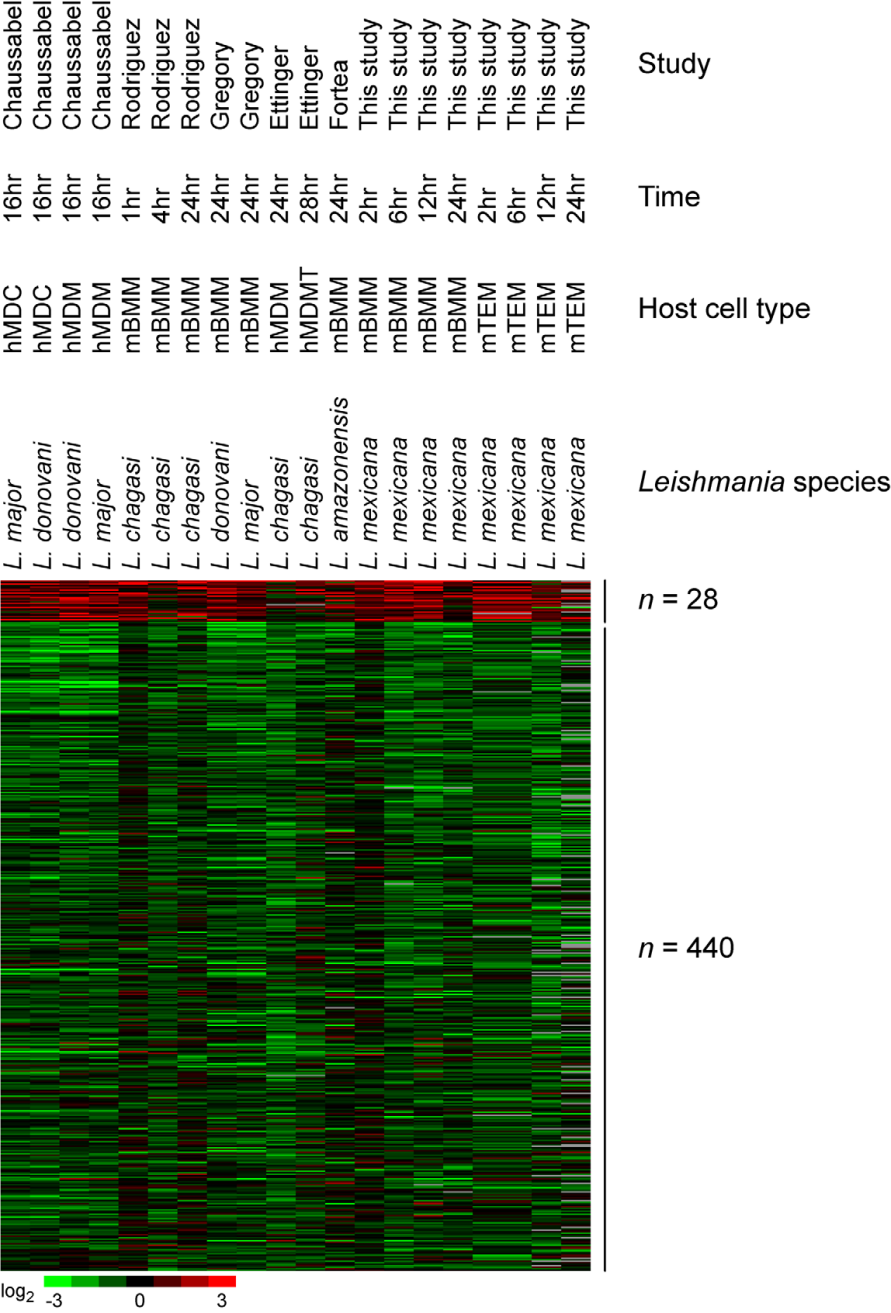
Comparison of transcriptional response to *Leishmania* infection compiled from this study and previous microarray studies. Meta-analysis was performed on the transcriptional response to *Leishmania* infection using data from this study and 5 additional studies. The heatmap shows genes upregulated (n = 28) and downregulated (n = 440) by *Leishmania* species. List of genes are shown in Dataset S2. hMDC, human monocyte-derived dendritic cells; hMDM, human monocyte-derived macrophages; mBMM, mouse bone marrow-derived macrophages; hMDMT, human monocyte-derived macrophages and T cells; mTEM, mouse thioglycollate-elicited macrophages.

**Figure 3 pntd-0000648-g003:**
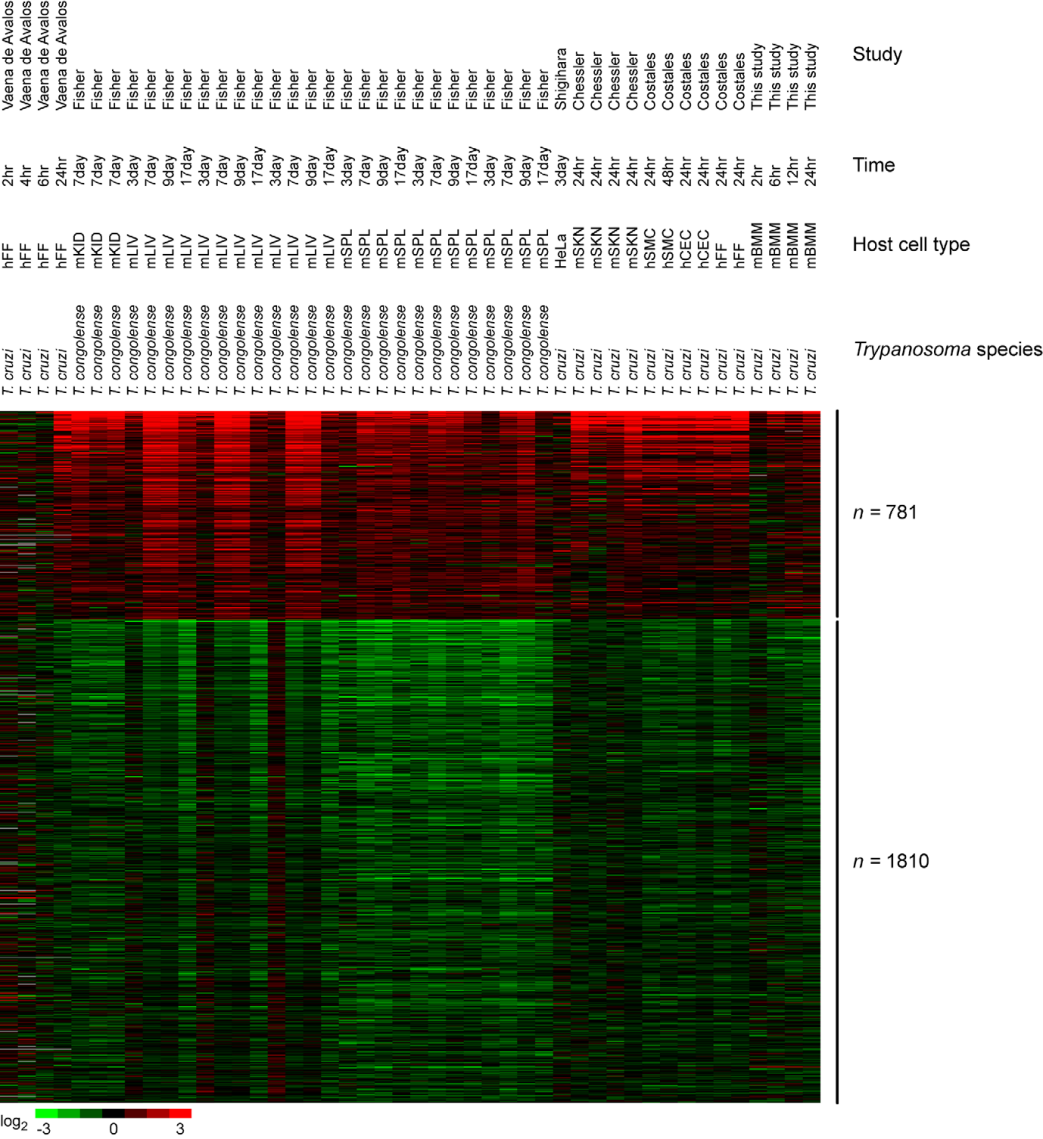
Comparison of transcriptional response to *Trypanosoma* infection compiled from this study and previous microarray studies. Meta-analysis was performed on the transcriptional response to *Trypanosoma* infection using data from this study and 5 additional studies. The heatmap shows genes upregulated (n = 781) and downregulated (n = 1810) by *Trypanosoma* species. List of genes are shown in Dataset S3. hFF, human foreskin fibroblasts; mKID, mouse whole kidney; mLIV, mouse whole liver; mSPL, mouse whole spleen; HeLa, HeLa cells; mSKN, mouse whole skin; hSMC, human vascular smooth muscle cells; hCEC, human cardiac microvascular endothelial cells; mBMM, mouse bone marrow-derived macrophages.

Previous studies have characterized the transcriptional response to several *Leishmania* [13],[14],[16],[19],[20] and *Trypanosoma* [12],[15],[17],[21],[22] species using a variety of *in vitro* and *in vivo* infection models. In order to compare our macrophage derived transcriptional profiling data to previous studies, we performed a “meta-analysis” of all publicly available expression profiling studies of the host response to *Leishmania* and *Trypanosoma* species (Figures 2 and 3). This analysis showed that the transcriptional responses of macrophages to *L. mexicana* and *T. cruzi* observed in this study showed commonality with the transcriptional responses to *Leishmania* and *Trypanosoma* species observed in other studies, despite the important differences in the species of parasites and the types of mammalian cells characterized.

Specifically, we found that all *Leishmania* species produced a transcriptional signature in host cells characterized by very small numbers of upregulated genes (n = 28) and a much larger number of downregulated genes (n = 440) (Figure 2 and Dataset S2). This observation suggests that infection by *Leishmania* species may have a suppressive effect on host transcription. Furthermore, the ability to infect the host cell in a very “silent” manner, causing minimal induction of host genes and enabling *Leishmania* to establish infection in the host macrophage and remain protected from the host immune response, may be a phenomenon common to multiple *Leishmania* species.

Our meta-analysis indicated that the transcriptional response to *Trypanosoma* species was far more robust than the response to *Leishmania* species with 781 genes upregulated and 1810 genes downregulated (Figure 3 and Dataset S3). This was the result of analyzing *in vivo* as well as *in vitro* experiments. The transcriptional responses to *in vivo* infection by *Trypanosoma congolense* (causative agent of bovine African Trypanosomiasis) [15] showed many commonalities with the transcriptional response to *in vitro* infection by *Trypanosoma cruzi* [12],[17],[21],[22]. This was surprising since *T. cruzi* is an intracellular pathogen that replicates within the host cell cytoplasm, while *T. congolense* is an extracellular pathogen that replicates in the blood stream. It has previously been reported that *T. brucei*, an etiological agent of human African Trypanosomiasis that is related to *T. congolense*, can activate macrophages via direct stimulation through its Variable Surface Glycoprotein (VSG) as well as via induction of host IFNG [29],[30],[31]. This may partially explain the similarity in host transcriptional response to *T. congolense* and *T. cruzi*.

In order to determine the biological function of genes induced or suppressed by the kinetoplastid pathogens, gene ontology analysis was performed. Very few biological processes were over-represented at a statistically significant level by the genes induced or suppressed by *Leishmania* species. The only over-represented biological process among genes upregulated by *Leishmania* was intracellular signalling cascade (Table 3). The two processes over-represented in genes downregulated by *Leishmania* were electron transport and oxidative phosphorylation (Table 4).

**Table 3:** Biological processes induced by *Leishmania* and *Tryapanosoma* species as determined by multi-study meta-analysis.

Biological processes induced by protozoan pathogens			
Pathogen	p-value	Biological process	Genes
*T. cruzi*	3.58E-19	Immunity and defense	*Abcc4, Angptl4, B2m, C3ar1, Ccl2, Ccl5, Ccl7, Ccr1, Ccr2, Cd47, Cd69, Cd86, Cebpb, Chek1, Chek2, Ctss, Cxcl1, Cxcl16, Cxcl2, Cxcl9, Dph2, F10, Fcer1g, Fos, Gadd45b, Gbp1, Gbp4, Gca, Gsg2, Hla-dma, Hla-e, Hspa1b, Hspa5, Hspb8, Icam1, Ier3, Ifi16, Ifi30, Ifi35, Ifit1, Ifit2, Ifitm1, Ifitm3, Il15, Il18, Il1a, Il1b, Il1rn, Il4r, Irf1, Irf7, Irf8, Isgf3g, Klf6, Klrg1, Lair1, Lgals1, Lgals3, Lgals3bp, Lgals9, Litaf, Lrrc59, Lrrc8c, Mocos, Myd88, Ncf2, Nfil3, Nmi, Nod1, Oasl, Parp3, Pbef1, Pla2g4a, Pla2g7, Plscr1, Ppid, Ppp2r2a, Ppp3cc, Ptgs2, Ptpn22, Pvr, Pvrl2, Rnpep, Samhd1, Slamf8, Slc11a1, Slfn5, Sod2, Stat2, Stat3, Tap1, Tap2, Tapbp, Tbk1, Tcirg1, Thbs1, Tnf, Tnfaip2, Tnfrsf1b, Was, Xbp1*
	1.56E-08	Cytokine and chemokine mediated signaling pathway	*Ccl2, Ccl5, Ccl7, Ccr1, Ccr2, Cx3cl1, Cxcl1, Cxcl2, Cxcl9, Il15, Il18, Il1a, Il1b, Il1rn, Il4r, Osm4, Ptpn2, Sirpa, Tlr1, Tlr2, Tlr3, Tlr4, Tnf, Tnfrsf1b, Traip*
	2.65E-08	Interferon-mediated immunity	*Cxcl9, Gbp1, Gbp4, Ifi16, Ifi35, Ifit1, Ifit2, Irf1, Irf7, Irf8, Isgf3g, Nmi, Oasl, Slamf8, Stat2*
	1.19E-03	Macrophage-mediated immunity	*Cxcl1, Cxcl16, Cxcl2, Cxcl9, Gbp1, Gbp4, Il1a, Il1b, Lgals3bp, Litaf, Stat2, Tnfaip2*
	1.68E-03	Cytokine/chemokine mediated immunity	*Ccl2, Ccl5, Ccl7, Ccr1, Ccr2, Cxcl9, Il1a, Il1b, Tbk1, Tnfaip2, Tnfrsf1b*
	5.32E-03	Granulocyte-mediated immunity	*Ccr1, Ccr2, Cd47, Cd69, Cxcl1, Cxcl2, Gca, Ncf2*
	1.18E-02	T-cell mediated immunity	*B2m, Cd69, Cd86, Ctss, Cxcl1, Cxcl2, Hla-dma, Hla-e, Ifi30, Ifi35, Nmi, Slamf8, Slfn5, Tapbp, Tcirg1*
	2.28E-02	JAK-STAT cascade	*Ifi35, Il15, Il18, Il4r, Jak2, Nmi, Ptpn2, Stambp, Stat2, Stat3*
	2.69E-02	DNA replication	*Cdc6, Fen1, Gins1, Hmgn3, Lig1, Mcm3, Mcm4, Mcm5, Mcm6, Orc6l, Pole, Prim2a, Rfc3, Rfc4, Rfc5, S100a11, Top2a, Wrn*
	3.00E-02	Ligand-mediated signaling	*Ccl2, Ccl5, Ccl7, Cd86, Cxcl1, Cxcl2, Cxcl9, Edg5, Grn, Il15, Il18, Il1a, Il1b, Il1rn, Il4r, Lair1, Pbef1, Slc1a3, Tnf*
	4.79E-02	Other immune and defense	*Cxcl9, Gsg2, Lgals1, Lgals3, Lgals9, Lrrc59, Lrrc8c, Myd88, Nod1, Pla2g4a, Pla2g7, Ptpn22, Rnpep, Tnfaip2*
*L. mexicana*	1.30E-02	Intracellular signaling cascade	*Cxcl1, Cxcl2, Dusp1, Gadd45g, Jak3, Pik3c2a, Stam2*

**Table 4:** Biological processes suppressed by *Leishmania* and *Tryapanosoma* species as determined by multi-study meta-analysis.

Biological processes repressed by protozoan pathogens			
Pathogen	p-value	Biological process	Genes
*T. cruzi*	2.69E-04	Lipid, fatty acid and steroid metabolism	*Aacs, Abca3, Abcd3, Acad8, Acadm, Acadsb, Acadvl, Acbd4, Acly, Acot11, Acox1, Acp6, Acsl1, Adipor1, Adipor2, Agpat2, Apoe, Ascc1, Atp11c, C2orf43, C7orf10, Cav1, Cav2, Cdipt, Chkb, Cmas, Cpt2, Crat, Crot, Cyb5a, Cyp2e1, Dci, Decr2, Dgat1, Dgka, Ebpl, Echdc3, Elovl5, Ephx1, Fasn, Fdx1, Gba2, Gcdh, Gpam, Gpx1, Gpx4, Habp4, Hadh, Hadha, Hmgcl, Hmgcs2, Hpgd, Hsd17b4, Hsdl1, Impad1, Ivd, Kiaa0274, Lpgat1, Lpin1, Mcat, Mgll, Mmd, Mmd2, Mtmr3, Nudt3, Nudt4, Osbpl2, Osbpl5, Osbpl9, Pccb, Pcyt1a, Pcyt2, Pex19, Phyh, Pik4ca, Pip5k1c, Plscr4, Pmvk, Ppap2b, Ppapdc2, Ppara, Prkab1, Prkab2, Prkag1, Pten, Rnpepl1, Sacm1l, Sc5dl, Scap, Scarb1, Sec14l2, Slc27a4, Slc37a4, Sorl1, Srebf1, Srebf2, St3gal2, Stard10, Stard4, Sult1c1, Tmem23, Tns1, Usf2*
	1.85E-02	Fatty acid metabolism	*Aacs, Acad8, Acadm, Acadsb, Acadvl, Acot11, Acox1, Acsl1, Adipor1, Adipor2, Cpt2, Crat, Crot, Cyp2e1, Dci, Decr2, Echdc3, Elovl5, Fasn, Gcdh, Hadh, Hadha, Hmgcl, Ivd, Mcat, Mmd, Mmd2, Pccb, Phyh, Prkag1, Rnpepl1, Slc27a4*
	1.91E-02	Amino acid metabolism	*Acy1, Adi1, Aga, Akap13, Aldh5a1, Aldh6a1, Arhgef12, Arhgef18, Arhgef3, Asnsd1, Bcat2, Bckdhb, Bckdk, Cbs, Ccbl1, Cpt2, Crat, Crot, Csad, Ctbp1, Dph5, Fah, Fahd1, Fahd2a, Fasn, Fbx08, Glud1, Glul, Grhpr, Hibadh, Ilvbl, Kmo, Kynu, Me1, Nadsyn1, Nfs1, Papss1, Papss2, Qdpr*
*L. mexicana*	1.25E-03	Electron transport	*Atp5j2, Blvra, Cat, Cox5b, Cox6a1, Cox7a2l, Cyb5r1, Ndufa2, Ndufa5, Ndufa6, Ndufa9, Ndufb5, Ndufb8, Ndufs3, Ndufv1, Sdha, Sdhb, Sdhc, Tbxas1, Txn2, Uqcr, Uqcrb, Uqcrc1, Uqcrc2, Uqcrh*
	3.25E-03	Oxidative phosphorylation	*Atp5j2, Cox5b, Cox6a1, Ndufa2, Ndufa5, Ndufa6, Ndufa9, Ndufb5, Ndufb8, Ndufs3, Ndufv1, Sdha, Sdhb, Uqcr, Uqcrb, Uqcrh*

Genes upregulated by *Trypanosoma* species were involved in a number of immune-related biological processes, including immunity and defense, interferon-mediated immunity, and macrophage-mediated immunity (Table 3). This is consistent with the immune-related nature of *T. cruzi*-induced genes identified in this study (Table 2). Genes downregulated by *Trypanosoma* species were involved with several metabolic processes including lipid, fatty acid and steroid metabolism (Table 4).

### Distinct signatures of classical, alternative and deactivation of macrophages

**Figure 4 pntd-0000648-g004:**
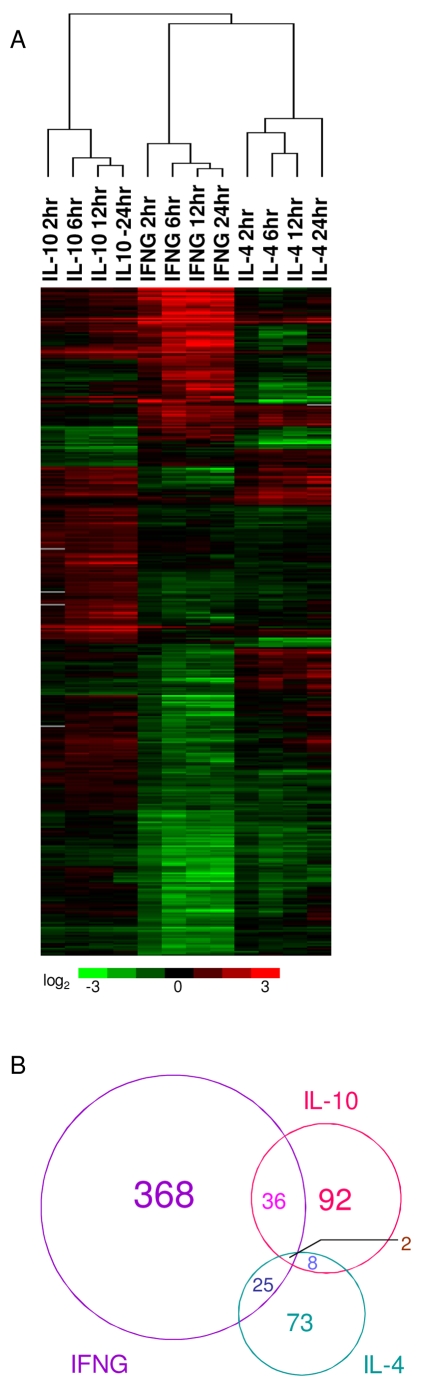
Comparison of transcriptional responses to classical activation, alternative activation, and macrophage deactivation. A. Unsupervised two-dimensional cluster analysis was performed on genes exhibiting statistically significant variability between the three conditions, as determined by multiclass SAM (n = 1489). B. The Venn diagram depicts the overlap of genes significantly upregulated, as determined by pairwise SAM analysis to unstimulated controls, by IFNG, IL-4, IL-10, both IFNG and IL-4, both IFNG and IL-10, both IL-4 and IL-10, and all three cytokines.

In addition to activation through engagement of PRRs, macrophages can also be activated by various cytokines. We therefore compared pathogen recognition programs to cytokine mediated activation programs in macrophages in order to assess the relationships between the infections and activation states. To first compare the relationship between classical activation, alternative activation, and deactivation of macrophages, we activated BMM with IFNG, IL-4, or IL-10, respectively, and compared the transcriptional profiles of these cells to that of untreated macrophages over a 24 h time course. Genes displaying significant changes in response to the three cytokine treatments were identified by performing multiclass analysis using SAM, and similarities in activation patterns were emphasized by hierarchical clustering. We found that IFNG, IL-4, and IL-10 produced distinct activation profiles in macrophages (Figure 4A). An analysis of genes significantly upregulated by IFNG, IL-4, or IL-10 in pairwise SAM analyses compared to untreated macrophages showed that the three cytokines induced mostly non-overlapping sets of genes (Table 5). Out of the 431 genes significantly induced by IFNG, only 38 were also induced by IL-10, and only 27 were also induced by IL-4 (Figure 4B). IL-10 and IL-4 only induced 10 of the same genes out of the 138 genes induced by IL-10 and the 108 genes induced by IL-4. The three sets shared only 2 genes in common.

**Table 5:** Comparison of genes induced by IFNG, IL-4, and IL-10.

Expression ratio (log2) of specific genes induced by one cytokine only			
Cytokine	>3	2–3	1–2
IFNG	*Ccl2, Ccl7, Cd69, Gbp3, Gbp5, Gbp6, Ifi44, Ifit2, Igtp, Mnda, Rgs1, Serpina3f, Slamf8, Slco3a1*	*Acsl1, B230207M22Rik, Ccl5, Ccnd2, Ccrl2, Cd83, Cd86, Cmpk2, Ctsc, Cxcl1, Cxcl10, D14Ertd668e, Denr, Ets2, Fcgr4, Fzd7, Gch1, Gdap10, Ggct, Herc5, I830012O16Rik, Ifi204, Ifih1, Ifit1, Ifit3, Il1rn, Irgm1, Irgm2, Jdp2, Lmo4, Mx1, Nod1, Oasl2, Parp14, Phf11, Pla2g16, Rnf213, Rsad2, Slamf7, Slc7a11, Slfn4, Slfn5, Stx11, Tnf, Tnfaip3, Usp18, Wars*	*0610038D11Rik, 1110002E22Rik, 1110038F14Rik, 1190002H23Rik, 1200003I10Rik, 1200009I06Rik, 2010106G01Rik, 2210012G02Rik, 2310007H09Rik, 2310016C08Rik, 2310058D17Rik, 2410039M03Rik, 4921509J17Rik, 4930403L05Rik, 4930427A07Rik, 5031414D18Rik, 9030607L20Rik, 9130209A04Rik, 9230105E10Rik, 9930023K05Rik, A930033M14Rik, AA960436, AI451617, Acsl5, Adar, Agfg1, Ak3l1, Ampd3, Ankrd57, Arhgef3, Armc8, Arrdc4, Asns, Ass1, Atf4, Atp8a2, B4galt3, B4galt5, BB123696, BC006779, BC013712, BC046404, Bambi-ps1, Basp1, Bat2d, Bcl2a1b, Bcl2a1c, Bfar, Birc2, Birc3, Bmi1, Bst2, Car13, Ccdc25, Ccl3, Cd1d1, Cdc42ep2, Chac1, Chmp4b, Ciita, Clcn7, Clec2d, Clec4e, Creb1, Creb5, Crem, Csnk1d, Csprs, Cxcl16, Cxcl2, Cybb, Daxx, Dcp2, Ddit3, Ddx58, Ddx60, Dhx58, Dio2, Dlk1, Dock4, Dtx3l, Dusp16, Dusp28, Egr1, Ehd1, Eif2ak2, Emr1, Enc1, Etnk1, Fabp3, Fam102b, Fam46c, Fam82a2, Farp2, Fas, Fndc3a, Foxred1, Glipr2, Glrp1, Gna13, Gnaq, Gpd2, Gpr141, Gtf2h1, Gtpbp2, Gvin1, H2-Q7, H2-T10, H2-T23, H2-T24, Haghl, Herc3, Hk1, Hk2, Hspa2, Icam1, Ifi203, Ifi205, Ifi35, Ifrd1, Il12rb2, Il15, Il15ra, Il27, Inpp5b, Insig1, Irak2, Irf8, Isg20, Itgav, Jak2, Katna1, Kdr, Kitl, Lass6, Lcp2, Lgals9, Lpar1, Lrrc14, Lrrc8c, Ltbp1, Ly6c1, M6pr, Mafk, Mdk, Mdm2, Mfsd7a, Mitd1, Mlkl, Mmp14, Mobkl1a, Mov10, Mpzl1, Mt2, Mtmr14, Mx2, Myd88, Nampt, Nfkb1, Nfkbie, Nmi, Nod2, Nr3c1, Nrp2, Nsbp1, Nt5c3, Oas1b, Oas1g, Oas2, Oasl1, Obfc2a, Ogfr, Olfr319, Olfr635, Otud1, P4ha1, Parp12, Parp9, Pcgf5, Pcmtd1, Pde4b, Peli1, Pfkfb3, Pfkp, Phlda1, Phlpp, Pla2g4a, Plaur, Plk2, Pml, Pmm2, Poldip3, Ppa1, Ppap2a, Ppap2b, Ppfibp2, Ppm1k, Ppp1r15b, Ppp2r2a, Prkx, Prpf38a, Psat1, Psmb10, Psmb9, Pstpip2, Ptafr, Rab11fip1, Rab12, Rab20, Ralgds, Rasa4, Rasgef1b, Rassf1, Rbm7, Rgl1, Rnf114, Rnf135, Rnf14, Rnf34, Samd9l, Samhd1, Sco1, Sema3c, Serpinb1a, Sestd1, Sgk1, Slc15a3, Slc2a6, Slc30a1, Slc31a1, Slc31a2, Slc3a2, Slc43a3, Slc6a9, Slfn1, Slfn2, Smpdl3b, Snx20, Soat2, Sp100, Sp110, Spata13, Spred1, Spred2, Spty2d1, Srgn, Srxn1, St3gal1, St3gal5, St6galnac4, St7, Stam2, Stat1, Stat2, Stk19, Stoml1, Stradb, Stx2, Tank, Tap1, Tapbp, Tapbpl, Tbc1d9, Tgm2, Tgoln1, Tgs1, Tm9sf4, Tmcc3, Tmem132a, Tmem2, Tnfrsf1a, Tnfrsf1b, Tnfsf9, Tor3a, Tpm4, Traf1, Trafd1, Trex1, Trib3, Trim13, Trim21, Trim30, Trim34, Trps1, Ttc39b, Ttc9c, Tyk2, Ubash3b, Ubd, Ube2l6, Ube2z, Ugcg, Usp12, Wdr20b, Wdr37, Wnt9a, Xkr8, Zbtb5, Zc3h12c, Zc3hav1, Zc3hc1, Zcchc2, Zfp429, Zfp800, Znfx1, Zufsp, Zyx*
IL-4		*Atp6v0d2, Egr2*	*1810011H11Rik, 2410042D21Rik, Acad8, Ak2, Arfgap2, Atic, Atp6v0a1, Baiap2, Batf3, Bcar3, Brd4, Brwd1, C030015D21Rik, Casp6, Chst7, Ctdp1, Cyp20a1, Daglb, Dusp4, ENSMUSG00000055697, Fam63a, Fchsd2, Fem1c, Flcn, Fyn, Herpud1, Herpud2, Hsph1, Il11ra1, Il1rl2, Il6st, Ipmk, Itgax, Itgb1, Lsm14b, Mat2a, Med1, Med15, Mettl9, Mgl1, Mgl2, Mllt3, Mybbp1a, Nat13, Necap1, OTTMUSG00000016703, P2ry1, Pcyt2, Pde12, Peo1, Pnpla8, Pparg, Ppp2r3a, Prosc, Prpf19, Prps1, Psmc4, Ptcd2, Rab15, Raly, Rnf181, Rragd, Sh3kbp1, Slc25a13, Slc30a4, Slc39a8, Tmco3, Tmed9, Tmem144, Ubqln1, Wdr45l*
IL-10	*Ednrb, Il4ra*	*Gda, Tnfsf14*	*4930435H24Rik, 4930471M23Rik, 5430427O19Rik, 6430527G18Rik, AB124611, Abcg1, Acat2, Aldh3b1, Aldoa, Ap1b1, Apoc2, Auh, BB031773, Bcl3, C1qb, C1qc, Ccl6, Ccr5, Cd74, Cdk2ap2, Cdkn2d, Cebpb, Coro1a, Cox7a2l, Ctsa, Cyth4, EG625174, Enpp1, Eps8, Fabp4, Fcgr1, Fcgr3, Fdps, Flot1, Fxc1, Fxyd2, Gbp2, Gcnt2, H2-DMa, H2-gs10, Hist1h3a, Hlx, Hmox1, Hsd17b10, Htra1, Ifitm2, Ifitm6, Il21r, Kif3a, Lilrb4, Lrrc25, Ly6e, Mafb, Malat1, Mefv, Mmp19, Mmp8, Mvd, Naaa, Ndrg1, Nfil3, Olfm1, Pdpn, Pld3, Plek, Pltp, Ppil2, Psme1, Ptpn1, Rac2, Sbno2, Sirpb1a, Smox, Spint1, Tcirg1, Tgfbi, Tha1, Timp1, Tle3, Tlr1, Tm2d2, Tmem49, Tmem8, Tnfaip8l2, Trim46, Tspan13, Tspo, Zic4*
**Specific genes induced by more than one cytokine (expression ratio varies)**			
IFNG and IL-4		*Arg2, Cd274, Ch25h, Cish, Clic4, Csf1, Flrt2, Flt1, Fmnl2, Gigyf2, Klf4, Maea, Mmp13, Psmd2, Rai12, Rars, Rel, Ripk2, Rnf19b, Snn, Socs1, Stam, Tlr4, Vegfc, Yme1l1*	
IFNG and IL-10		*1200016E24Rik, 4933412E12Rik, Axud1, Btg1, Casp4, Ccl12, Ccl4, Cd40, Cdkn1a, F10, Fam26f, Fgl2, Flrt3, Gadd45b, Gsdmd, H2-Aa, H2-Ab1, H2-Eb1, Ier3, Ifi47, Igf2bp2, Il18bp, Il1b, Irf7, Ly6a, Ly6f, Mcoln2, Mxd1, Nfkbiz, Pgs1, Psmb8, Saa3, Slc28a2, Socs3, Stat3, Zfp36*	
IL-4 and IL-10		*Csf2rb, Dhrs9, Fcgr2b, Mrc1, Ncoa4, Ptgs1, Rab3il1, Tcfec*	
IFNG and IL-4 and IL-10		*Irf1, Pim1*	

As expected, IFNG induced a large cluster of interferon-stimulated genes that were not induced by IL-4 or IL-10 (e.g. *Ccl2*, *Ccl7*, *Gbp3*, *Gbp5*, *Gbp6*, *Ifi44*, *Ifit2*, *Ifi204*, *Ifih1*, *Ifit1*, *Ifit3*, *Ifi203*, *Ifi205*, *Ifi35*, *Isg20*, *Stat1*, *and Stat2*). IL-4 induced a number of novel or unclassified genes (*Atp6v0d2*, *1810011H11RIK*, *2410042D21RIK*, *Rnf181*, *Tmem144*) and genes not previously associated with alternative activation (*Erg2*, *Casp6*, *Chst7*, *Daglb*, *Il1rl2*, *Rab15*, *Raly*). For example, the PPAR binding protein (*Pparbp/Med1*) was induced by IL-4 and likely interacts with PPARG to act as a co-activator for this nuclear receptor. Diacylglycerol lipase beta (*Daglb*) could be involved in metabolic regulation by alternatively activated macrophages [32].

IL-10 induced its own distinct transcriptional signature in macrophages. Notable genes induced only by IL-10 include *Il4ra*, *Ccl6*, *Il21r*, *Mmp8*, *Mmp19*, *Timp1*, *and Tlr1* (Table 5). Surprisingly, there were more genes (n = 38) induced in common by both IL-10 and IFNG (eg. *Casp4*, *Ccl12*, *Ccl4*, *Irf7*, *Ly6a*, *Socs3*, *Ifi47*, *Stat3*) than genes (n = 10) induced by both IL-10 and IL-4 (eg. *Dhrs9*, *Fcgr2b*, *Ptgs1*, *Tcfec*) (Figure 4B). However, one of the genes induced by IL-10 is *Il4ra*, which is consistent with a previous study that suggested exposure to IL-10 enhanced responsiveness to IL-4 [33]. Also of note is the induction of *Il21r*. IL-21 receptor shows significant sequence and structural homology to IL-4 receptor alpha and has been shown to augment alternative macrophage activation [34], further suggesting that IL-10 may indirectly promote alternative activation by increasing sensitivity to IL-4, IL-13 and IL-21.

In order to determine what biological processes and pathways are induced by each of the cytokines, we performed gene ontology analysis on genes upregulated by only one cytokine as well as on genes upregulated by more than one cytokine (Table 6). Gene ontology analysis of the genes induced by IL-4 alone did not reach statistical significance for any biological process classification. This is likely due to the smaller number of genes in this category as well as the fact that many of these genes are novel and have unknown functions, highlighting the need for additional work on IL-4 signaling in macrophages and its associated gene expression patterns. In contrast to IL-4 treatment, IL-10 and IFNG treatment produced unique gene lists that were enriched in the biological processes of immunity and defense and cytokine and chemokine mediated signaling pathways (Table 6). IL-10 activated genes were also enriched in lipid and fatty acid transport, while IFNG activated genes were enriched in interferon mediated immunity, T cell-mediated immunity, ligand-mediated signaling, signal transduction, macrophage-mediated immunity, and apoptosis (Table 6). As noted above, IFNG and IL-10 induced genes that fall into similar categories and were quite distinct from IL-4 induced genes.

**Table 6:** Comparison of biological processes induced by IFNG, IL-4, and IL-10.

Biological processes induced by one or more cytokine			
	P value	Biological process	Genes
**IFNG only**	4.63E-18	Interferon-mediated immunity	*Cxcl10, Gbp3, Gbp5, Gbp6, Ifi203, Ifi205, Ifi35, Ifit1, Ifit2, Ifit3, Irf8, Nmi, Oas1b, Oas1g, Oas2, Oasl1, Oasl2, Slamf7, Slamf8, Stat2*
	5.23E-16	Immunity and defense	*C2ta, Ccl2, Ccl3, Ccl5, Ccl7, Ccrl2, Cd1d1, Cd69, Cd86, Clec2d, Clec4e, Cxcl1, Cxcl10, Cxcl16, Cxcl2, Dusp16, Fcgr3a, Gbp2, Gbl5, Gbl6, H2-D4, H2-Q7, H2-T24, Haghl, Hspa2, Icam1, Ifi203, Ifi205, Ifi35, Ifit1, Ifit2, Ifit3, Il12rb2, Il15, Il15ra, Il1rn, Irak2, Irf8, Lgals9, Lrrc8c, Myd88, Nfkb1, Nmi, Nod1, Nod2, Oas1b, Oas1g, Oas2, Oasl1, Oasl2, Pbef1, Phlpp, Pla2g4a, Plaur, Ppp2r2a, Ptafr, Rgs1, Samhd1, Slamf7, Slamf8, Slfn5, Stat2, Tap1, Tapbp, Tapbpl, Tnf, Tnfrsf1a, Tfnrsf1b*
	6.48E-06	T-cell mediated immunity	*C2ta, Cd1d1, Cd69, Cd86, Cxcl1, Cxcl2, H2-D4, H2-Q7, H2-T24, Ifi35, Il15ra, Nmi, Slamf7, Slamf8, Slfn5, Tapbp, Tapbpl, Tnfrsf1a*
	4.49E-05	Ligand-mediated signaling	*Ccl2, Ccl3, Ccl5, Ccl7, Cd86, Cxcl1, Cxcl10, Cxcl2, Edg2, Il12rb2, Il15, Il15ra, Il1rn, Kitl, Mdk, Pbef1, Tnf, Tnfrsf1a, Wnt9a*
	7.97E-05	Cytokine and chemokine mediated signaling pathway	*Ccl2, Ccl3, Ccl5, Ccl7, Ccrl2, Cxcl1, Cxcl10, Cxcl2, Fas, Il12rb2, Il15, Il15ra, Il1rn, Tnf, Tnfrsf1a, Tnfrsf1b, Traf1*
	2.34E-04	Signal transduction	*0710001B24Rik, 2010206G01Rik, 9130017C17Rik, Arhgef3, Ccl2, Ccl3, Ccl5, Ccl7, Ccrl2, Cd86, Creb1, Creb5, Crem, Csnk1d, Csprs, Cxcl1, Cxcl10, Cxcl2, Dock4, Dusp16, Edg2, Emr1, Ets2, Fabp3, Fas, Fcgr3a, Gna13, Gnag, Gpr141, Hrasls3, Hrb, Icam1, Ifi35, Il12rb2, Il15, Il15ra, Il1rn, Inpp5b, Irak2, Jak2, Kdr, Kitl, Lcp2, Ltbp2, M6pr, Mdk, Myd88, Nfkb1, Nmi, Nrp2, Ogfr, Olfr319, Pbef1, Pde4b, Plk2, Ppap2a, Ppap2b, Ppm1k, Prkx, Pstpip2, Ptafr, Rab12, Rab20, Ralgds, Rasa4, Rasgef1b, Rassf1, Rgl1, Rgs1, Rnf14, Sema3c, Sgk, Spata13, Stam2, Stat2, Tnf, Tnfrsf1a, Tnfrsf1b, Traf1, Trib3, Tyk2, Wnt9a*
	2.55E-03	Macrophage-mediated immunity	*Clec4e, Cxcl1, Cxcl10, Cxcl16, Cxcl2, Fcgr3a, Gbp3, Gbp5, Gbp6, Stat2, Tnfrsf1a*
	2.74E-02	Cytokine/chemokine mediated immunity	*Ccl2, Ccl3, Ccl5, Ccl7, Ccrl2, Cxcl10, Tnfrsf1a, Tnfrsf1b*
	2.98E-02	Apoptosis	*Arhgef3, Bcl2a1c, Birc2, Birc3, D11Lgp2e, Ddx58, Fas, Ifih1, Il15, Jak2, Lgals9, Mdm2, Nfkb1, Nod1, Nod2, Rassf1, Sgk, Tnf, Tnfrsf1a, Traf1, Ube2z*
**IL-10 only**	1.20E-05	Immunity and defense	*C1gb, C1gc, Ccl6, Ccr5, Cd74, Cebpb, Fcgr1, Fcgr3, Gbp2, H2-DMa, H2-Q5, Ifitm2, Ifitm6, Il21r, Il4ra, Nfil3, Ppil2, Tcirg1, Tnfaip8l2, Tnfsf14*
	7.47E-03	Cytokine and chemokine mediated signaling pathway	*Ccl6, Ccr5, Il4ra, Ptpn1, Sirpb1, Tlr1, Tnfsf14*
	3.40E-02	Lipid and fatty acid transport	*Abcg1, Apoc2, Fabp4, Pltp, Tspo*
**IFNG and IL-10**	1.04E-06	Immunity and defense	*Ccl12, Ccl4, Cd40, F10, Gadd45b, H2-Aa, H2-Ab1, H2-Eb', H2-T23, Ier3, Il1b, Irf7, Saa3, Stat3*
	1.67E-03	MHCII-mediated immunity	*H2-Aa, H2-Ab1, H2-Eb1*
	5.47E-03	T-cell mediated immunity	*Cd40, H2-Aa, H2-Ab1, H2-Eb1, H2-T23*
	1.52E-02	Apoptosis	*Axud1, Casp4, Gadd45b, Il1b, Socs3, Stat3*

### IFNG activation signature in macrophages is more similar to TNF than IFNB

TNF and IFNB contribute to the early inflammatory cytokine milieu and have both been shown to induce classical macrophage activation [1]. In contrast to IFNG, which is produced mainly by lymphocytes, TNF and IFNB are cytokines that are often produced at the very early acute stages of an immune response by many other cell types [35],[36]. While the IFNs initiate a signalling cascade that involves the JAK family of tyrosine kinases and STAT family of transcription factors [37],[38], TNF signals through TRAFs to activate the transcription factors NF-kB and AP-1 [39],[40]. Signalling via the IL-17 receptor has also been shown to occur through the adaptor molecule TRAF6 leading to the activation of NF-kB and AP-1, suggesting it may activate macrophages in a manner similar to TNF [41],[42],[43].

To determine the macrophage activation signatures following stimulation with TNF, IFNB, and IL-17 and their relationship with IFNG, macrophages were activated by IFNB, TNF and IL-17. To identify genes upregulated and downregulated by activation through these cytokines, the resulting data was analyzed by SAM via two-class unpaired statistical comparisons against untreated control macrophages. To compare gene expression changes between all of the different cytokines, multiclass SAM analysis followed by hierarchical clustering analysis was performed on the expression profiles of macrophages activated by IFNG, IFNB, TNF, and IL-17.

**Figure 5 pntd-0000648-g005:**
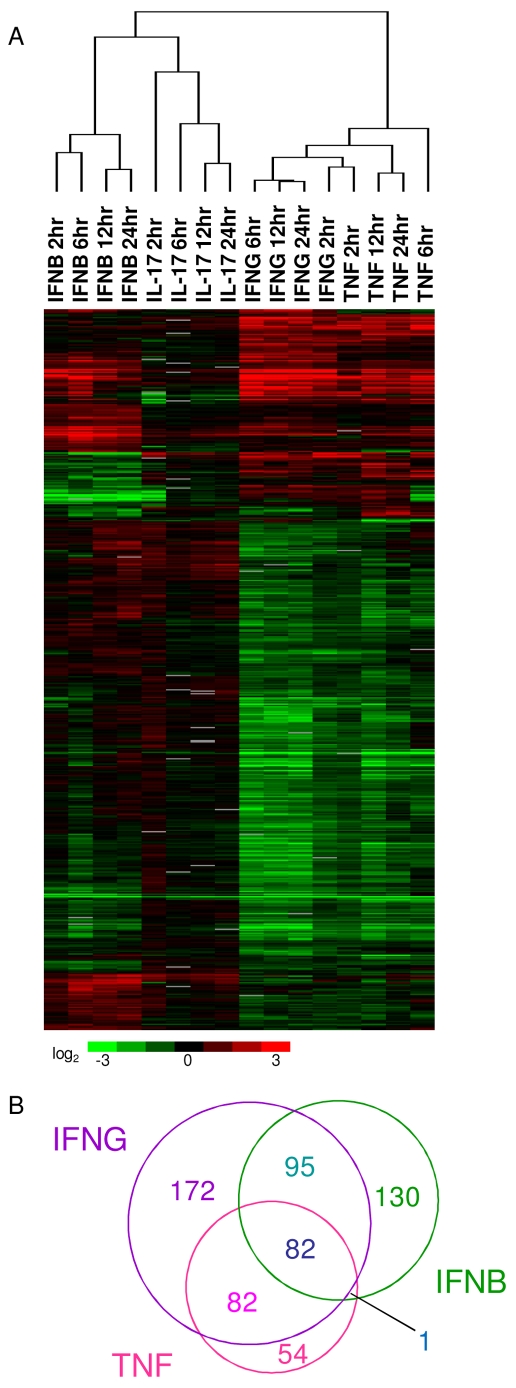
Comparison of transcriptional responses to cytokines implicated in classical macrophage activation. A. Unsupervised two-dimensional cluster analysis was performed on genes exhibiting statistically significant variability between stimulation with IFNG, IFNB, TNF, and IL-17, as determined by multiclass SAM (n = 773). B. The Venn diagram depicts the overlap of genes significantly upregulated, as determined by pairwise SAM analysis to unstimulated controls, by IFNG, IFNB, TNF, both IFNG and IFNB, both IFNG and TNF, both IFNB and TNF, and all three cytokines.

We found that the response to IFNG was most related to the response to TNF, whereas IFNB and IL-17 produced more distinct expression profiles (Figure 5A). This was surprising given that the interferons signal through overlapping JAK-STAT pathways, while TNF signals through a distinct NF-kB pathway. An analysis of the genes induced by TNF revealed a number of interferon-stimulated genes, suggesting that TNF may be inducing expression of an interferon by macrophages. This is consistent with a previous study which showed that TNF induces IFNB production in macrophages [44]. To determine whether TNF induced IFNB, we measured the amount of IFNB mRNA in TNF-stimulated cells by qPCR. TNF-stimulated macrophages upregulated IFNB transcript starting at 2 h after stimulation and peaking at 6 h after stimulation (Figure S7 A). Although this explained why TNF induced the expression of interferon-stimulated genes, it did not explain why the macrophage response to TNF is more similar to the response to IFNG than to IFNB. Further analysis of the array data revealed that IFNG stimulation induced expression of TNF (Tables 5 and 7), while IFNB stimulation did not. To confirm this finding, we measured the expression of TNF in cells stimulated by IFNG or IFNB by qPCR. We found that IFNG induced significant levels of TNF transcript by 2 h after stimulation, while IFNB failed to induce TNF expression (Figure S7 B). In addition, we found through cytometric bead analysis (CBA) of culture supernatants that IFNG-stimulated macrophages secreted high levels of TNF protein by 6 h after stimulation (Figure S7 C). The level of TNF peaked at 12 h post-stimulation at approximately 400 pg/mL. These selective interactions between cytokine signalling pathways explain the similarity in gene expression observed for cytokines with disparate signalling pathways.

**Table 7:** Comparison of genes induced by IFNG, IFNB, and TNF.

Expression ratio (log2) of specific genes induced by one cytokine only			
Cytokine	>3	2–3	1–2
IFNG	*Ccl2, Ccl7*	*Cd83, Ly6a, Wars*	*0610038D11Rik, 1110038F14Rik, 1190002H23Rik, 1200009I06Rik, 2210012G02Rik, 2310007H09Rik, 2310016C08Rik, 4921509J17Rik, 4930403L05Rik, 4930427A07Rik, 5031414D18Rik, 9030607L20Rik, 9130209A04Rik, 9930023K05Rik, A930033M14Rik, Acsl5, Agfg1, Ak3l1, Arhgef3, Armc8, Arrdc4, Atp8a2, B4galt5, BB123696, BC046404, Basp1, Bat2d, Birc2, Bmi1, Bst2, Btg1, Ccdc25, Ccl3, Cdkn1a, Chmp4b, Ciita, Clcn7, Creb1, Creb5, Crem, Csnk1d, Cxcl16, Cxcl2, Cybb, Ddit3, Dlk1, Dock4, Egr1, Emr1, Enc1, Etnk1, Fam102b, Fmnl2, Foxred1, Gadd45b, Gigyf2, Glipr2, Gna13, Gsdmd, Gtf2h1, Gtpbp2, H2-Aa, H2-Ab1, H2-Eb1, H2-T23, H2-T24, Haghl, Hk2, Hspa2, Ier3, Ifrd1, Igf2bp2, Il12rb2, Il1b, Il27, Inpp5b, Irak2, Irf8, Itgav, Klf4, Lass6, Lcp2, Lrrc14, Lrrc8c, Ly6c1, Maea, Mobkl1a, Mt2, Mtmr14, Myd88, Nfkb1, Nr3c1, Nrp2, Nsbp1, Obfc2a, Olfr319, P4ha1, Pcmtd1, Pde4b, Pfkp, Pgs1, Phlpp, Plaur, Pmm2, Poldip3, Ppp2r2a, Prpf38a, Psat1, Psmd2, Ptafr, Rai12, Ralgds, Rars, Rassf1, Rbm7, Saa3, Sco1, Sema3c, Serpinb1a, Sestd1, Sgk1, Slc31a1, Slc3a2, Slc43a3, Smpdl3b, Snn, Snx20, Spata13, Spred2, Spty2d1, Srgn, St3gal5, St6galnac4, St7, Stam, Stat3, Stk19, Stoml1, Stradb, Stx2, Tapbp, Tapbpl, Tbc1d9, Tgm2, Tgoln1, Tlr4, Tm9sf4, Tmem132a, Tmem2, Tnfrsf1b, Trps1, Ttc9c, Ubash3b, Ubd, Ugcg, Wdr20b, Wdr37, Wnt9a, Xkr8, Yme1l1, Zbtb5, Zc3hav1, Zc3hc1, Zfp36, Zfp429, Zfp800, Zyx*
IFNB		*Fcgr1*	*100040620, 1110018G07Rik, 1700016D18Rik, 1810035L17Rik, 2810474O19Rik, 4932438A13Rik, 5-Mar, 5430427O19Rik, 5730508B09Rik, 6330442E10Rik, 6820401H01Rik, A230046K03Rik, Aadacl1, Aftph, Aipl1, Aldh1l2, Ankle2, Apol9a, Apol9b, Arid4a, Asb13, Ascc3, Asxl3, Atp10a, Atp11b, Axl, B2m, BB031773, Bco2, C330011K17Rik, C330023M02Rik, Ccdc86, Ccdc90a, Cdkal1, Chd6, Cnp, Ctla2b, Cycs, Dhh, Dhrs9, E130102H24Rik, Endod1, Epsti1, Erap1, Fam46a, Fbxw17, Fem1c, Frmd4a, Gcnt2, Glcci1, Gng11, H2-T22, H2-T9, Hfe, Hmgn3, Ier5, Il18, Ilk, Iqwd1, Irf2, Irf9, Itpr1, Kat2b, Kif5c, Klra8, Kpna4, Lamp2, Ly6e, Ly86, Mllt3, Ms4a6b, Ms4a6c, Mtcp1, Nub1, OTTMUSG00000016703, Oas1c, Oas3, Olfr843, P2ry14, P2ry6, Papd4, Pi4kb, Plekhf2, Pltp, Pnpt1, Pols, Rab9, Rabep1, Rin2, Rnf139, Rnf31, Rtp4, S1pr2, Sdc3, Serpinb1c, Serpinb6b, Sgcb, Sgk3, Slamf9, Slc2a8, Slfn10, Smc5, Snw1, Spock2, Stxbp3a, Sumf2, Taok3, Tfb2m, Timeless, Tlr3, Tlr7, Tlr8, Tmem184b, Tmem209, Tor1aip1, Tor1aip2, Tpst1, Tspan13, Uba7, Usf1, Usp25, Usp42, Vcpip1, Zc3hav1l, Zcchc6, Zfp281, Zfp295, Zfp319, Zfp455*
TNF		*Ednrb, Gbp2*	*4833445I07Rik, 9030425E11Rik, 9130221J17Rik, Abcc1, Ahr, C730045O03Rik, Cav1, Chpf, Clec5a, Coq10b, Ddit4, Dnmt3l, Dusp4, Egr2, Ext1, Gbe1, Gss, Gtf2a1, Herpud1, Hmox1, Irak3, Jag1, Lox, M6pr-ps, Mafg, Mfap3l, Nqo1, Olfr1272, Olfr214, Orai2, Osbpl3, Pcdh7, Pim3, Prdx6-rs1, Prkar2b, Psmd10, Psmd11, Reln, Rps6ka2, Slc11a1, Slpi, Src, Syk, Tcfec, Tiparp, Ttc39c, Txnrd1, Ugt1a6a, Unc5b, Vasp, Vcan, Zfp719*
**Specific genes induced by more than one cytokine (expression ratio varies)**			
IFNG and IFNB	*1110002E22Rik, 2310058D17Rik, 4933412E12Rik, 9230105E10Rik, AA960436, Adar, B4galt3, BC006779, BC013712, Bambi-ps1, Bfar, Ccl12, Ccnd2, Cd86, Cish, Clec2d, Csprs, Ctsc, Daxx, Dcp2, Dhx58, Dusp28, Eif2ak2, Fam26f, Fam82a2, Farp2, Fcgr4, Fgl2, Flt1, Fndc3a, Fzd7, Gch1, Gnaq, H2-Q7, H2-T10, Hk1, Ifi205, Irf7, Katna1, Kdr, Kitl, Ltbp1, Ly6f, Mafk, Mdk, Mitd1, Mlkl, Mov10, Nmi, Nod1, Oas1b, Oas2, Ogfr, Olfr635, Otud1, Parp12, Parp9, Pcgf5, Peli1, Pfkfb3, Pml, Ppa1, Ppm1k, Ppp1r15b, Prkx, Psmb10, Psmb8, Psmb9, Rasa4, Rgl1, Rnf114, Rnf135, Rnf19b, Rnf34, Samd9l, Samhd1, Serpina3f, Slamf8, Slc28a2, Slc30a1, Slfn1, Sp110, Stat2, Tap1, Tgs1, Tmcc3, Tnfrsf1a, Tor3a, Trim21, Trim30, Ttc39b, Tyk2, Usp12, Znfx1, Zufsp*		
IFNG and TNF	*1200003I10Rik, 1200016E24Rik, 2410039M03Rik, Acsl1, Ampd3, Ankrd57, Asns, Ass1, Atf4, Bcl2a1b, Bcl2a1c, Birc3, Car13, Cd1d1, Cdc42ep2, Chac1, Clec4e, Cxcl1, Denr, Dusp16, Ehd1, Ets2, F10, Fas, Flrt3, Gdap10, Ggct, Gpd2, Gpr141, Herc3, Icam1, Ifi47, Insig1, Jdp2, Lmo4, Lpar1, M6pr, Mcoln2, Mdm2, Mfsd7a, Mmp14, Mpzl1, Nampt, Nfkbie, Nfkbiz, Nod2, Phlda1, Pim1, Pla2g4a, Ppap2a, Ppap2b, Ppfibp2, Pstpip2, Rab11fip1, Rab12, Rab20, Rel, Rnf14, Rsad2, Slc2a6, Slc31a2, Slc6a9, Slc7a11, Slfn2, Soat2, Socs3, Spred1, Srxn1, St3gal1, Stam2, Stx11, Tank, Tnf, Tnfaip3, Tnfsf9, Tpm4, Traf1, Trib3, Trim13, Ube2z, Vegfc, Zc3h12c*		
IFNB and TNF	*Mthfr*		
IFNG and IFNB and TNF	*2010106G01Rik, AI451617, Arg2, Axud1, B230207M22Rik, Casp4, Ccl4, Ccl5, Ccrl2, Cd274, Cd40, Cd69, Ch25h, Clic4, Cmpk2, Csf1, Cxcl10, D14Ertd668e, Ddx58, Ddx60, Dio2, Dtx3l, Fabp3, Fam46c, Flrt2, Gbp3, Gbp5, Gbp6, Glrp1, Gvin1, Herc5, I830012O16Rik, Ifi203, Ifi204, Ifi35, Ifi44, Ifih1, Ifit1, Ifit2, Ifit3, Igtp, Il15, Il15ra, Il18bp, Il1rn, Irf1, Irgm1, Irgm2, Isg20, Jak2, Lgals9, Mmp13, Mnda, Mx1, Mx2, Mxd1, Nt5c3, Oas1g, Oasl1, Oasl2, Parp14, Phf11, Pla2g16, Plk2, Rasgef1b, Rgs1, Ripk2, Rnf213, Slamf7, Slc15a3, Slco3a1, Slfn4, Slfn5, Socs1, Sp100, Stat1, Trafd1, Trex1, Trim34, Ube2l6, Usp18, Zcchc2*		

There was a significant overlap between the genes up-regulated by IFNG, IFNB, and TNF (Figure 5B). Of the 219 genes induced by TNF, 164 were also induced by IFNG, consistent with the observation that IFNG induced TNF production by macrophages (Figure S7 B–C) as described above. Genes induced by both TNF and IFNG included *Cxcl1, Ifi47, Mmp14, Nod2, Socs3, and Tnf* (Table 7). IFNG and IFNB also up-regulated many of the same genes (n = 177) including *Ccl12, Ifi205, Irf7, Nod1, and Stat2* (Table 7). A subset of these genes (n = 82) were induced by all three of the cytokines, including *Ccl4, Ccl5*,*Gbp3, Gbp5, Gbp6, Ifi203, Ifi204, Ifi35, Ifi44, Ifih1, Ifit1, Ifit2, Ifit3, Irf1, Isg20, Mmp13, Socs1, and Stat1* (Table 7). A number of genes were induced by only one cytokine; for example, the pattern recognition receptors *Tlr3*, *Tlr7*, and *Tlr8* are only induced by IFNB and not by TNF or IFNG.

Gene ontology analysis was performed on genes up-regulated by only one cytokine as well as on genes up-regulated by more than one cytokine. IFNG, TNF, and IFNB function in many of the same biological processes, including immunity and defense, interferon-mediated immunity, and macrophage-mediated immunity (Table 8). Although most of the enriched biological processes are shared between the three cytokines, a few are specific to individual cytokines. For example, only IFNG induced genes enriched for MHCII-mediated immunity (Table 8). TNF induced genes enriched for several unique processes such as neurogenesis and ectoderm development. IFNG and IFNB both induced genes in the biological processes of proteolysis and protein metabolism and modification, but TNF did not (Table 8).

**Table 8:** Comparison of biological processes induced by IFNG, IFNB, and TNF.

Biological processes induced by one or more cytokine			
	P value	Biological process	Genes
**IFNG only**	3.81E-07	Immunity and defense	*C2ta, Ccl2, Ccl3, Ccl7, Cxcl16, Cxcl2, Gadd45b, H2-Aa, H2-Ab1, Hd-D4, H2-Eb1, H2-T24, Haghl, Hspa2, Ier3, Il12rb2, Il1b, Irak2, Irf8, Lrrc8c, Maea, Myd88, Nfkb1, Phlpp, Plaur, Ppp2r2a, Ptafr, Saa3, Stat3, Tapbp, Tapbpl, Tnfrsf1b*
	1.71E-03	T-cell mediated immunity	*C2ta, Cxcl2, H2-Aa, H2-Ab1, H2-D4, H2-Eb1, H2-T23, H2-T24, Tapbp, Tapbpl*
	3.76E-03	MHCI-mediated immunity	*H2-D4, H2-T23, H2-T24, Tapbp, Tapbpl*
	6.54E-03	MHCII-mediated immunity	*C2ta, H2-Aa, H2-Ab1, H2-Eb1*
**IFNB only**	3.09E-05	MHCI-mediated immunity	*B2m, H2-Q5, H2-T22, H2-T9, Hfe*
	6.83E-03	T-cell mediated immunity	*B2m, H2-Q5, H2-T22, H2-T9, Hfe, Slamf9*
**TNF only**	9.11E-04	Neurogenesis	*Ednrb, Egr2, Jag1, Pcdh7, Reln, Rps6ka2, Src, Unc5b*
	1.74E-03	Ectoderm development	*Ednrb, Egr2, Jag1, Pcdh7, Reln, Rps6ha2, Src, Unc5b*
	2.01E-03	Immunity and defense	*9030425E11Rik, Abcc1, Ahr, Clec5a, Cog10b, Gbp2, Irak3, Jag1, Prdx6-rs1, Slc11a1, Src, Syk*
**IFNG and IFNB**	1.97E-05	Interferon-mediated immunity	*Irf7, Nmi, Oas1b, Oas2, Slamf8, Stat2*
	2.50E-03	Immunity and defense	*Ccl12, Cd86, Cish, Clec2d, Fcgr3a, H2-Q7, Irf7, Nmi, Nod1, Oas1b, Oas2, Samhd1, Slamf8, Stat2, Tap1, Tnfrsf1a*
	2.91E-02	Proteolysis	*9230105E10Rik, Ctsc, Pml, Psmb10, Psmb8, Psmb9, Rnf135, Rnf34, Serpina3f, Trim21, Trim30, Trim34, Usp12*
	3.66E-02	Protein metabolism and modification	*0710001B24Rik, 9230105E10Rik, B4galt3, Ctsc, Eif2ak, Flt1, Ibrdc3, Katna1, Kdr, Mlkl, Mov10, Pml, Ppm1k, Prkx, Psmb10, Psmb8, Psmb9, Rnf135, Rnf34, Serpina3f, Tor3a, Trim21, Trim30, Trim 34, Tyk2, Usp12*
**IFNG and TNF**	1.60E-03	Apoptosis	*Bcl2a1c, Birc3, Fas, Mdm2, Nod2, Rel, Socs3, Tnf, Traf1, Ube2z*
	3.23E-03	Inhibition of apoptosis	*Bcl2a1c, Birc3, Mdm2, Rel, Socs3, Ube2z*
	1.24E-02	Signal transduction	*Cxcl1, Dusp16, Edg2, Ets2, Fas, Flrt3, Gpr141, Icam1, M6pr, Pbef1, Ppap2a, Ppap2b, Pstpip2, Rab12, Rab20, Rel, Rnf14, Socs3, Stam2, Tnf, Traf1, Trib3, Vegfc*
**IFNG and IFNB and TNF**	1.49E-20	Interferon-mediated immunity	*Cxcl10, Gbp3, Gbp5, Gbp6, Ifi203, Ifi35, Ifit1, Ifit2, Ifit3, Irf1, Oas1g, Oasl1, Oasl2, Slamf7*
	9.42E-14	Immunity and defense	*Ccl4, Ccl5, Ccrl2, Cdc274, Cd40, Cd69, Csf1, Cxcl10, Gbp3, Gbp6, Ifi203, Ifi35, Ifit1, Ifit2, Ifit3, Il15, Il15ra, Il1rn, Irf1, Lgals9, Oas1g, Oasl1, Oasl2, Rgs1, Slamf7, Slfn5*
	2.48E-06	Cytokine and chemokine mediated signaling pathway	*Ccl4, Ccl5, Ccrl2, Cd40, Csf1, Cxcl10, Il15, Il15ra, Il1rn, Socs1*
	2.43E-03	T-cell mediated immunity	*Cd274, Cd490, Cd69, Ifi35, Il15ra, Slamf7, Slfn5*
	1.95E-02	Macrophage-mediated immunity	*Csf1, Cxcl10, Gbp3, Gbp5, Gbp6*
	4.37E-02	Apoptosis	*Axud1, Casp5, Ddx58, Ifih1, Il15, Jak2, Lgals9, Ripk2*

Unexpectedly, IL-17 produced minimal transcriptional changes in comparison to the other cytokines. Only 7 genes were positively regulated based on pairwise SAM analysis against unstimulated controls (Table 9). Although several of the upregulated genes were genes also induced by IFNG, IFNB, and TNF (e.g. *Cxcl1*, *Oasl2*, *Phf11*), the magnitude of the upregulation was much lower. In order to determine whether the paucity of transcriptional responses to IL-17 was due to the lack of IL-17 receptor expression on BMMs, we stained BMMs with an anti-IL-17 receptor antibody for analysis by flow cytometry (Figure S8). We found that BMMs do express the IL-17 receptor, suggesting that transcriptional responses to IL-17 may require additional co-stimulation or pre-stimulation by another cytokine or antigen.

**Table 9:** Genes altered by IL-17 stimulation of bone marrow-derived macrophages.

Upregulated by IL-17		
Gene Name	Gene Symbol	Fold change
Early growth response 1	*Erg1*	3.291448
chemokine (C-X-C motif) ligand 1	*Cxcl1*	2.568791
2′-5′ oligoadenylate synthetase-like 2	*Oasl2*	2.419333
PHD finger protein 11	*Phf11*	2.135998
Ring finger protein 213	*Rnf213*	2.119956
beta-2 microglobulin	*B2m*	2.069215
ARP3 actin-related protein 3 homolog (yeast)	*Arp3*	2.041713

### Relationship between pathogen and cytokine mediated macrophage activation

**Figure 6:**
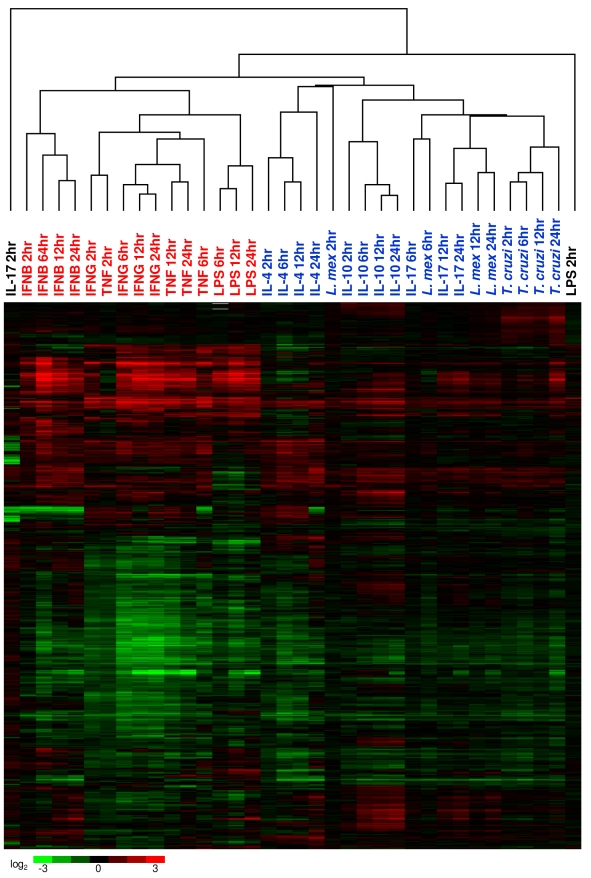
Comparison of transcriptional responses to cytokines and intracellular parasites. Unsupervised two-dimensional cluster analysis was performed on all pathogen and cytokine arrays, using genes exhibiting statistically significant variability between these conditions, as determined by SAM (n = 5414). Arrays in red text highlight the relationship between cytokines involved in classical macrophage activation and the bacterial antigen LPS. Arrays in blue text highlight the relationship between the protozoan pathogens *L. mexicana* and *T. cruzi* and the cytokines IL-4, IL-10, and IL-17.

The PAMPS expressed by intracellular protozoans such as *T. cruzi* and *L. mexicana* are much less well characterized than those of bacterial pathogens. Comparing the transcriptional responses of macrophages infected with kinetoplastids to those of macrophages stimulated by various cytokines may provide insights as to the types of receptors these pathogens engage and the signalling pathways they initiate. In order to compare the transcriptional changes associated with cytokine signalling and intracellular pathogen infection, cluster analysis was performed on all cytokine and pathogen arrays. This showed that innate activation by LPS was most closely related to classical activation by the cytokines IFNG and TNF (Figure 6). Activation by the cytokine IFNB was similar but not as closely related. This is consistent with a previous study showing that macrophage responses to LPS and immune complexes are more similar to classical activation than to alternative activation [45]. The similarity between LPS and TNF can also be attributed to the induction of *Tnf* in macrophages stimulated with LPS (Table 1). Furthermore, flow cytometry analysis showed that TNF was produced by approximately 15% of BMMs 4 h post-stimulation by LPS (Figure S9).

Innate macrophage activation by the protozoan pathogens branched separately from classical activation. Instead, macrophages infected by *T. cruzi* and *L. mexicana* clustered with macrophages stimulated by IL-17, IL-10, and IL-4. This suggests that infection by kinetoplastids results in a macrophage activation state that is more similar to alternative macrophage activation and macrophage deactivation than to classical macrophage activation. Although there are signs of an IFN response at later time points of *T. cruzi* infection, this signature is not strong enough to affect the overall clustering results.

### The transcriptional response of bone marrow-derived macrophages differs from that of identically treated thioglycollate-elicited macrophages

Historically, intra-peritoneal injection with Brewer's thioglycollate medium has been a convenient method to procure large numbers of macrophages for use in functional and biochemical studies [46]. More recently, bone marrow-derived macrophages have become widely used for such experiments. The transcriptional profile of these two types of macrophages may be divergent. Since all transcriptional profiling studies to date have been performed using a single type of macrophage, it is unknown how transcriptional responses may vary depending on the type of macrophage used.

**Figure 7:**
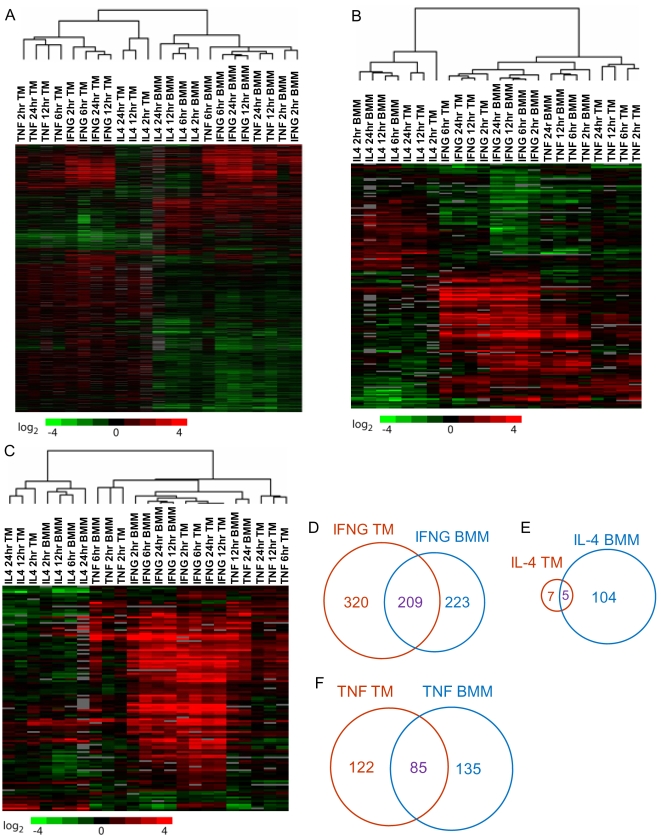
Differences in the transcriptional response in differentially derived macrophages. A. Unsupervised two-dimensional cluster analysis was performed on genes exhibiting statistically significant variability between the 6 groups (n = 3671): IFNG stimulation of bone marrow macrophages, TNF stimulation of bone marrow macrophages, IL-4 stimulation of bone marrow macrophages, IFNG stimulation of thioglycollate macrophages, TNF stimulation of thioglycollate macrophages, and IL-4 stimulation of thioglycollate macrophages. Arrays clustered based on the type of macrophage, not the type of cytokine. B. Multiclass SAM analysis was performed on arrays representing IFNG, TNF, and IL-4 stimulation of bone marrow macrophages. Cluster analysis was performed on genes exhibiting statistically significant differences amongst bone marrow derived macrophages only (n = 168). Arrays clustered based on the type of cytokine, not the type of macrophage. C. Multiclass SAM analysis was performed on arrays representing IFNG, TNF, and IL-4 stimulation of thioglycollate macrophages. Cluster analysis was performed on genes exhibiting statistically significant differences amongst thioglycollate macrophages only (n = 124). Arrays clustered based on the type of cytokine, not the type of macrophage. D. Genes induced by IFNG in thioglycollate-elicited macrophages (TM) were compared to genes induced by IFNG in bone marrow-derived macrophages (BMM). E. Genes induced by IL-4 in TMs were compared to genes induced by IL-4 in BMMs. F. Genes induced by TNF in TMs were compared to genes induced by TNF in BMMs.

To address this question, we treated BMMs and thioglycollate-elicited macrophages (TM) with the cytokines IFNG, TNF, and IL-4 and compared their transcriptional responses. We used hierarchical clustering analysis to identify the conditions in which the transcriptional responses were most similar. We found that arrays clustered based on the type of macrophage instead of the type of cytokine stimulation (Figure 7A). In order to determine whether this was due to differential expression of background transcripts or differential response to cytokine stimulation, we performed multiclass SAM analysis on only the bone marrow macrophage arrays. The genes identified from this analysis (n = 168) were then extracted from the thioglycollate macrophage dataset, and a hierarchical clustering analysis was performed (Figure 7B). We found that the arrays clustered based on the cytokine instead of the macrophage type, with the IFNG and TNF arrays clustering together and away from the IL-4 arrays irrespective of macrophage type. A similar result was obtained when the cluster analysis was performed on genes identified by a multiclass SAM analysis of only the thioglycollate arrays (n = 124) (Figure 7C). This shows that although the baseline transcriptional signatures of bone marrow-derived and thioglycollate-elicited macrophages are very different, the two types of macrophages respond to cytokines in a relatively similar fashion.

In order to determine more specific differences in gene induction in the two different types of macrophages, we analyzed genes upregulated by IFNG, TNF and IL-4 by pairwise SAM analysis to untreated controls for both thioglycollate and bone marrow-derived macrophages and compared the genes induced in the two types of macrophages (Figures 7D–F). Interestingly, a large number of genes induced by these cytokines were specific for the type of macrophage used. IFNG induced 209 genes in both TMs and BMMs, 320 genes only in TMs, and 223 genes only in BMMs (Figure 7D). Only five genes were induced in both TMs and BMMs by IL-4, while seven genes were induced only in TMs and 104 genes were induced only in BMMs (Figure 7E). TNF induced 85 genes in both TMs and BMMs, 122 genes only in TMs, and 135 genes only in BMMs (Figure 7F). Table 10 shows specific genes induced by these cytokines in one or both types of macrophages. These results suggest that TMs are somewhat more predisposed to classical activation whereas BMMs are more predisposed to alternative activation. Future work will define the basal differences between these two types of macrophages and determine why they respond differently to different forms of activation.

**Table 10:** Comparison of genes induced in bone marrow-derived versus thioglycollate-elicited macrophages.

	BMM and TM	BMM only	TM only
**IFNG**	*Ccl2, Ccl5, Ccl7, Cd40, Cd69, Cd83, Cd86, Cxcl10, Cxcl16, Fcgr4, Gbp3, Gbp5, Gbp6, Ifi203, Ifi205, Ifi35, Ifi47, Ifih1, Ifit2, Ifit3, Igf2bp2, Igtp, Il15ra, Il18bp, Il1rn, Il27, Irf1, Irgm, Jak2, Myd88, Nfkbie, Nfkbiz, Nmi, Nod1, Nod2, Oas1g, Oas2, Oasl1, Oasl2, Socs1, Socs3, Stat1, Stat2, Tap1, Tapbpl, Tnfaip3, Tnfrsf1a, Traf1* *	*Ccl12, Ccl3, Ccl4, Ccrl2, Cxcl1, Cxcl2, H2-Aa, H2-Ab1, H2-Eb1, H2-Q7, H2-T10, H2-T23, H2-T24, Ifi44, Ifit1, Il12rb2, Il15, Il1b, Irf7, Irf8, Isg20, Mmp13, Mmp14, Nfkb1, Stat3, Tnf, Tnfrsf1b, Tnfsf9* *	*Cxcl3, Cxcl9, Ifi27, Il10ra, Il12a, Il12rb1, Il13ra1, Il4ra, Irf2, Irf9, Isg15, Nfkb2, Nfkbia, Tlr1, Tnfsf13b* *
**IL-4**	*C030015D21Rik, Cish, Mmp13, Rab15, Rab3il1*	*Casp6, Cd274, Ch25h, Chst7, Daglb, Dhrs9, Dusp4, Fcgr2b, Il11ra1, Il1rl2, Il6st, Irf1Pparg, Socs1, Tlr4* *	*2610030P05Rik, B430119L13Rik, Chi3l3, Hist2h3c2, Mafb, Pdcd1lg2, Zcchc2*
**TNF**	*Ccl5, Cd69, Cxcl1, Gbp5, Gbp6, Ifi203, Ifih1, Ifit2, Ifit3, Il18bp, Irak3, Isg20, Jak2, Mmp13, Mx1, Mx2, Nfkbie, Nfkbiz, Nod2, Oasl2, Stat1, Tnfaip3, Tnfsf9, Traf1* *	*Ccl4, Ccrl2, Cd274, Cd40, Cxcl10, Gbp2, Gbp3, Ifi35, Ifi44, Ifi47, Ifit1, Igtp, Il15, Il15ra, Il1rn, Irf1, Mmp14, Oas1g, Oasl1, Socs1, Socs3, Tnf, Trafd1* *	*Ccl7, Cd14, Cd86, Cxcl3, Cxcl5, Ifi205, Il17ra, Il1a, Irf9, Isg15, Oas2, Tlr1, Traf5* *

*: Selected genes.

## Discussion

In this study, we have comprehensively evaluated the relationship between macrophage activation states induced by two intracellular protozoan parasites (*L. mexicana* and *T. cruzi*), a bacterial endotoxin (LPS), and various cytokines (IL-4, IL-10, IFNG, IFNB, TNF). We found that innate activation of mouse bone marrow-derived macrophages by LPS was most similar to classical macrophage activation by the cytokines IFNG and TNF. However, infection by the protozoan pathogens *T. cruzi* and *L. mexicana* elicited responses most similar to alternative activation by the Th2 cytokine IL-4 and to macrophage deactivation by the cytokine IL-10. Collectively, these results suggest that the macrophage activation state induced by kinetoplastid parasites is disparate from the activation state induced by bacterial PAMPs and Th1 cytokines.

The distinct signature of macrophage transcriptional responses to kinetoplastids is in contrast to a meta-analysis by Jenner and Young suggesting that immune cells respond to pathogens with a generalized common transcriptional program [47]. Jenner and Young compiled data from 32 published microarray studies, representing 77 different host-pathogen interactions. A cluster of 511 genes were identified that appeared to be co-regulated across the entire dataset, and these genes were termed the “common host response.” These transcripts were enriched for genes involved in the immune response against invading pathogens. In this study, we observed that the transcriptional response to the cytokines IFNG and TNF is highly related to the response to LPS. A meta-analysis of the microarrays from this study and the Jenner and Young “common host response” genes indicated that the cluster of genes shared by classical activation cytokines and LPS is highly related to the human common host response genes (Figure S10). This cluster includes many genes involved in the immune response, such as *Nfkb*, *Irf1*, *Irf7*, *Ifit1*, *Ifit2*, and *Myd88*. However, the transcriptional signature of early time points during kinetoplastid infection is clearly distinct from this common host response, highlighting again the difference between these protozoan parasites and bacterial pathogens.

The variability we found between pathogens/pathogen products likely reflect the differences in pattern recognition receptors utilized by each pathogen. Both protozoan pathogens induced fewer transcriptional responses than LPS, consistent with previous microarray studies comparing infection of macrophages by protozoan and non-protozoan pathogens [19]. However, *T. cruzi* induced a number of interferon stimulated genes by 24 h post-infection, consistent with a recent study by Chessler et al showing upregulation of interferon-stimulated genes in mice 24 h following intradermal infection with *T. cruzi* [22]. The upregulation of interferon-stimulated genes has been shown to be dependent on the induction of IFNB via signalling through a novel toll-like receptor-independent pathway [27],[28],[48].

The timing of the IFNB-induced gene upregulation in *T. cruzi*-infected macrophages is consistent with a previous study performed by de Avalos et al that implicated *T. cruzi* escape from a parasitophorous vacuole into the cytoplasm [21]. A possible explanation for the differences in responses to the two protozoan pathogens is that they reside in different intracellular compartments. *Leishmania* persists in a membrane-bound vacuole within the cell, while *T. cruzi* leaves this compartment within hours to freely replicate in the macrophage cytoplasm. Strikingly, infection by *L. mexicana* was so transcriptionally “silent” that the resulting activation profile was almost indistinguishable from uninfected cells. This is consistent with several other studies showing limited responses in various host tissues to various species of *Leishmania* [13],[14],[16],[19],[20]. One explanation for this silent infection could be that *Leishmania* shields itself from host detection by residing within the parasitophorous vacuole. Another explanation is that *L. mexicana* does not express any potent pattern recognition ligands and therefore cannot activate the host cell. A third explanation is that the parasite is actively suppressing host responses via binding of a host receptor or secretion of a virulence factor that interferes with the inflammatory response. Evidence for this explanation can be found in previous studies which have shown that *Leishmania* species can down-modulate macrophage responses using a variety of mechanisms [49],[50]. *L. mexicana* amastigotes have been shown to inhibit IL12 production by disrupting the NF-kB signalling pathway [51],[52]. This has been shown to downregulate the Th1 response in infected mice, leading to increased pathology [53]. Any one or a combination of these factors may play a role in the transcriptional silence of macrophages infected by *L. mexicana*.

The protozoan pathogens *T. cruzi* and *L.mexicana* produced transcriptional signatures in infected macrophages that were more closely related to alternative macrophage activation and macrophage deactivation than to classical macrophage activation. This lends support to studies showing that immunization with the immunodominant *T. cruzi* antigen, cruzipain, results in increased Th2 cytokine secretion [54] and increased arginase activity [55]. The induction of alternatively activated macrophages in *T. cruzi* infection results in persistent parasite growth within the cells [56], suggesting that *T. cruzi* may evade the host immune response by promoting alternative macrophage activation. The similarity in the transcriptional signatures resulting from *L. mexicana* infection and IL-10 stimulation is consistent with studies showing that *Leishmania* induces IL-10 during infection and that IL-10 plays an essential role in *Leishmania* pathogenesis [57],[58],[59]. These studies have demonstrated that IL-10 is induced via FcGR ligation by opsonized parasites [57],[59]. Hence, IL-10 may play a role in dampening the transcriptional response of macrophages to *Leishmania* infection.

Our analysis of macrophage responses to cytokine stimulation revealed stark differences in the activation profile of macrophages treated with IL-4 relative to macrophages treated with IFNG or IL-10 (Figure 2 and Table 5). A number of genes have been previously classified as markers of alternative macrophage activation [60], but were not identified in our analysis of genes induced by IL-4 (Table 5). These include *Ym1*/*Ym2* (*Chi3l3*/*Chi3l4*), *Fizz1/Relm-alpha* (*Retnla*), and *Arg1*. Because these genes are so specifically regulated by IL-4 and are not expressed under other conditions included in this study, they did not pass our pre-analysis filter of being ‘present in at least 70% of arrays’ and were not included in the final dataset used for statistical analysis by SAM (Dataset S1). This problem highlights a limitation of our analysis, in that genes that are expressed at very low levels and induced very specifically by individual cytokines may be filtered out of the overall analysis. When the data for these specific markers of alternative activation were extracted from the unfiltered dataset, they are clearly upregulated in IL-4 stimulated BMMs, as shown in Figure S6.

By conducting the experiments as time courses over 24 hours and grouping the time points for analysis, our statistical comparisons identify genes that are consistently up or down-regulated. Although important kinetic information is still preserved upon more detailed analysis (e.g. the identification of a late 24 h interferon response in *T. cruzi* infection), such detailed kinetic data cannot be extracted statistically due to the absence of sufficient replicates for each time point. Although we have added additional biological replicates to our kinetoplastid infections, resources have limited our ability to replicate the time courses for all of the different cytokines.

Despite the limitations described above, we have for the first time directly compared global gene expression profiles from macrophages activated by a diverse group of biologically important cytokines and pathogens. This dataset will be valuable to both the parasitology and macrophage biology communities as a resource for future experiments. Our data identified unique properties of classically activated, alternatively activated and deactivated macrophages as well as identified unexpected relationships between macrophage responses to pathogens and cytokines.

The functional phenotypes of macrophages in peripheral tissues depend on both their origin and the cytokine microenvironment to which they are exposed. In addition to the relationships between pathogen infection and cytokine stimulation, we have also evaluated the importance of macrophage origin on these transcriptional studies. The activation profiles of bone marrow derived macrophages were distinct from those of identically treated thioglycollate-elicited macrophages. Discrepancies between studies investigating macrophage activation can be at least partly attributed to the use of different types of macrophages. However, we observed that the two types of macrophage produced a similar transcriptional response to activation stimuli, indicating that although baseline gene expression in these macrophages is different, signalling and transcriptional responses are largely shared. Further understanding of the underlying mechanism for the differences between various types of macrophages will be important to the study of both macrophage biology and host-pathogen interactions.

## Supporting Information

Dataset S1 Dataset including all microarrays represented in Figures 1–6 of this study (dataset used for SAM analysis).
(5.74 MB ZIP)

Dataset S2 List of genes up- or down-regulated by *Leishmania* species.
(0.05 MB XLS)

Dataset S3 List of genes up- or down-regulated by *Trypanosoma* species.
(0.18 MB XLS)

Figure S1 Comparative analysis of RNA isolated from fresh versus frozen bone marrow derived macrophages. RNA from freshly prepared and cryopreserved BMMs were collected and hybridized post amplification against each other (cryopreserved BMM RNA labelled with Cy3 and fresh BMM RNA labelled with Cy5) on a MEEBO oligonucleotide array. The scatter plot shows the resulting median fluorescence intensities plotted on the X and Y axis for fresh and frozen macrophages. The correlation coefficient (R) is shown.
(1.02 MB PDF)

Figure S2 Purity of cultured bone marrow-derived macrophages (BMMs). The purity of bone marrow derived macrophages that were used in microarray experiments was confirmed by flow cytometry analysis using antibodies against CD11b and F4/80. (A) Histogram showing the percentage of BMMs (99.4%) stained with CD11b (filled) against unstained BMMs (unfilled). (B) Histogram showing the percentage of BMMs (93.8%) stained with F4/80 (filled) against unstained BMMs (unfilled).
(0.02 MB PDF)

Figure S3 Infection of BMM with *L. mexicana*. (A) Uninfected BMMs stained with Diff-Quik. (B) BMMs infected with *L. mexicana* at a MOI of 10 and stained with Diff-Quik 24 h post-infection. (C) BMMs infected with CFSE labelled *L. mexicana* at a MOI of 10, visualized by fluorescent microscopy. (D) Flow cytometry analysis on uninfected BMMs (D) and BMMs infected with CFSE-labelled *L. mexicana* at a MOI of 10 (E).
(0.49 MB PDF)

Figure S4 Induction of interferon-stimulated genes by *T. cruzi*. Quantitative real-time PCR analysis on cDNA from cells infected with *T. cruzi* or *L. mexicana* and from uninfected cells using primers directed against the interferon-stimulated genes IFIT3 (A) and IFI205 (B).
(0.01 MB PDF)

Figure S5 Comparative analysis of uninfected versus *T. cruzi* mock-infected BMMs. RNA from uninfected BMMs and BMMs treated with supernatant from uninfected BESM cells for 24 h (mock-infected BMMs) were collected and hybridized post-amplification against each other (uninfected BMM RNA labelled with Cy3 and mock-infected BMM RNA labelled with Cy5) on a MEEBO oligonucleotide array. The scatter plot shows the resulting median fluorescence intensities plotted on the X and Y axis for fresh and frozen macrophages. The correlation coefficient (R) is shown.
(0.88 MB PDF)

Figure S6 Induction of alternative macrophage activation markers by IL-4 stimulated BMMs. Heatmap showing the expression of genes that are known to be induced by IL-4 in alternatively activated macrophages, extracted from our IL-4 time course data. Black indicates unchanged level of expression relative to time 0 h, and red indicates upregulated levels expression.
(0.01 MB PDF)

Figure S7 Cross induction of classical activation cytokines. (A) Quantitative real-time PCR analysis of *Ifnb* expression in cells stimulated with recombinant TNF and on unstimulated cells. TNF induced production of *Ifnb* transcript by 6 h post-stimulation. (B) Quantitative real-time PCR analysis of *Tnf* expression in cells stimulated with recombinant IFNG and IFNB and on unstimulated cells. TNF induced production of *Ifnb* transcript by 6 h post-stimulation. IFNG induced expression of *Tnf* transcript by 2 h post-stimulation, but IFNB does not. (C) TNF protein secretion into the supernatant of IFNG-stimulated BMMs 6 h post treatment was measured by cytometric bead analysis (BD).
(0.01 MB PDF)

Figure S8 IL-17 receptor expression on bone marrow-derived macrophages Cell surface antigen staining was performed on BMMs using PE-conjugated IL-17R antibody. The histogram shows cells stained with IL-17R (filled) and cells stained with IgG2a isotype control (unfilled).
(0.01 MB PDF)

Figure S9 Induction of TNF by BMMs activated with LPS. Intracellular cytokine staining analysis of TNF production in unstimulated BMMs (A) and BMMs stimulated with 100 ng/uL of LPS for 4 h (B).
(0.02 MB PDF)

Figure S10 Relating mouse macrophage responses to the human “common host response” Heat map showing significantly altered genes as determined by multiclass SAM analysis for all cytokine and pathogen arrays (n = 5414). The graph on the right side represents the frequency that genes in the heat map appear in the human common host response set determined by Jenner and Young [47].
(0.06 MB PDF)

Methods S1 Methods for Supporting Information materials.
(0.06 MB DOC)
