# Supplementary figures and images for: Delineation of Diverse Macrophage Activation Programs in Response to Intracellular Parasites and Cytokines
Source: PLoS Negl Trop Dis. 2010 Mar 30;4(3):e648. doi: 10.1371/journal.pntd.0000648 (PMC2846935; doi:10.1371/journal.pntd.0000648)

Supplementary Figure 1

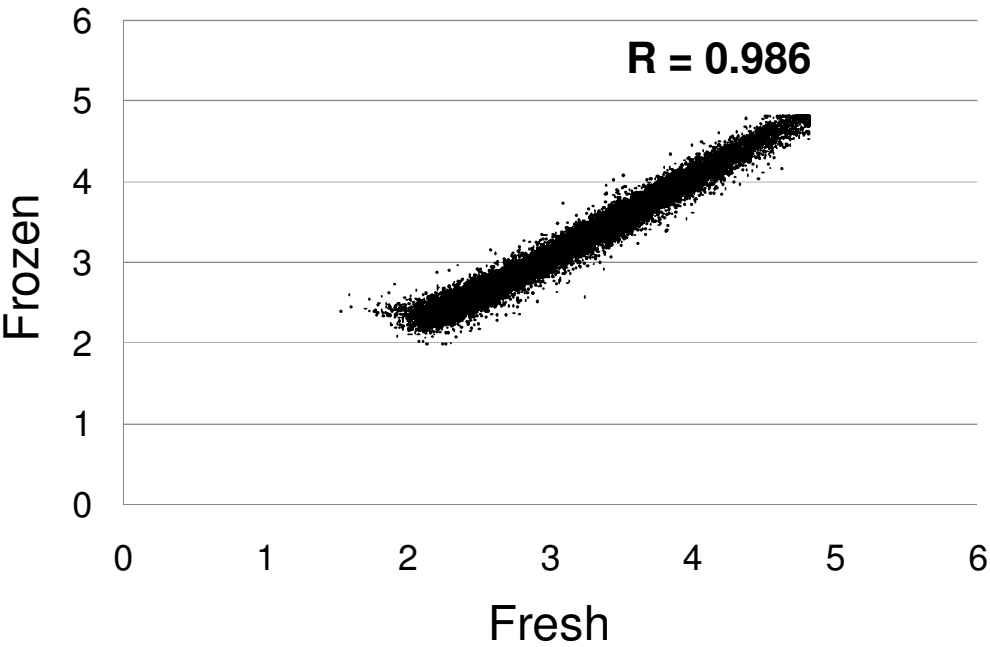

Supplement: Figure S1 — Comparative analysis of RNA isolated from fresh versus frozen bone marrow derived macrophages. RNA from freshly prepared and cryopreserved BMMs were collected and hybridized post amplification against each other (cryopreserved BMM RNA labelled with Cy3 and fresh BMM RNA labelled with Cy5) on a MEEBO oligonucleotide array. The scatter plot shows the resulting median fluorescence intensities plotted on the X and Y axis for fresh and frozen macrophages. The correlation coefficient (R) is shown. (1.02 MB PDF) [file pntd.0000648.s004.pdf]

Supplementary Figure 2

A

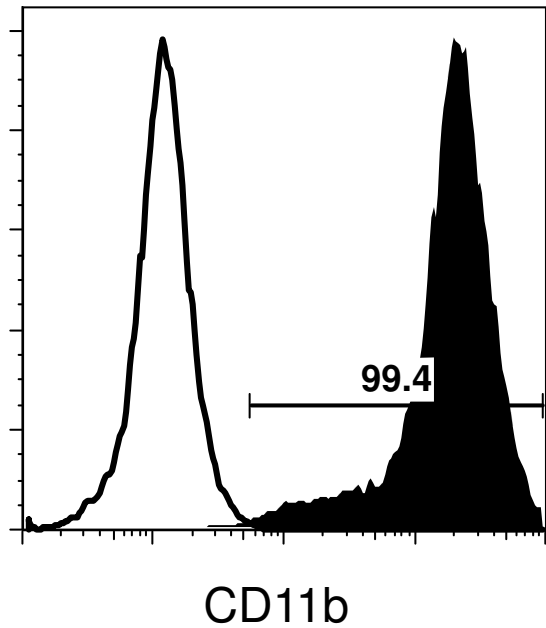

B

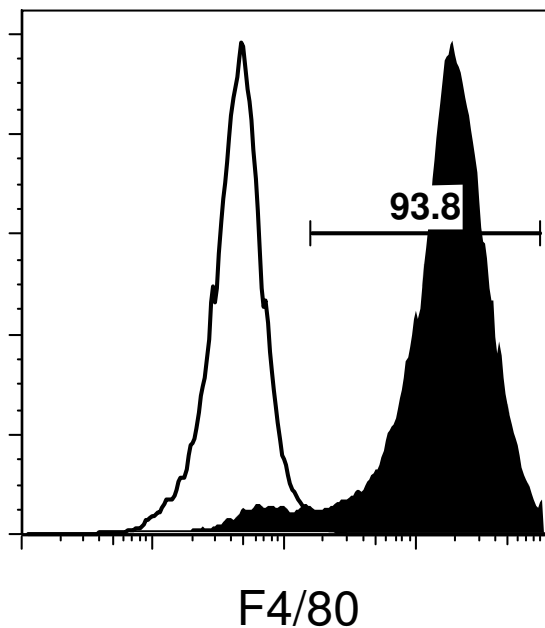

Supplement: Figure S2 — Purity of cultured bone marrow-derived macrophages (BMMs). The purity of bone marrow derived macrophages that were used in microarray experiments was confirmed by flow cytometry analysis using antibodies against CD11b and F4/80. (A) Histogram showing the percentage of BMMs (99.4%) stained with CD11b (filled) against unstained BMMs (unfilled). (B) Histogram showing the percentage of BMMs (93.8%) stained with F4/80 (filled) against unstained BMMs (unfilled). (0.02 MB PDF) [file pntd.0000648.s005.pdf]

Supplementary Figure 3

A

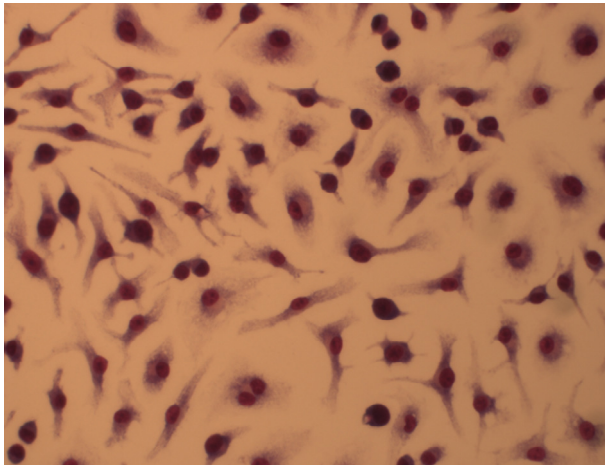

B

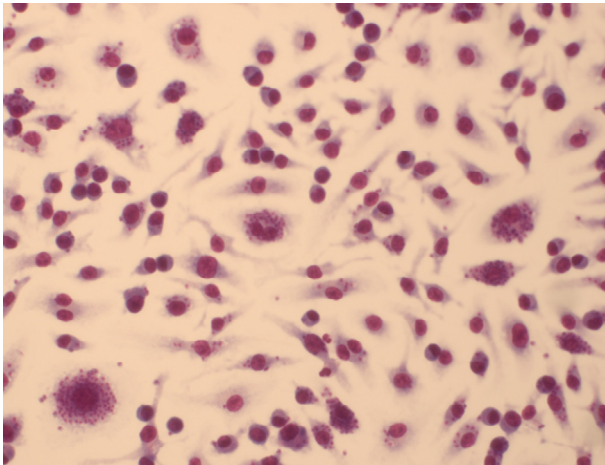

C

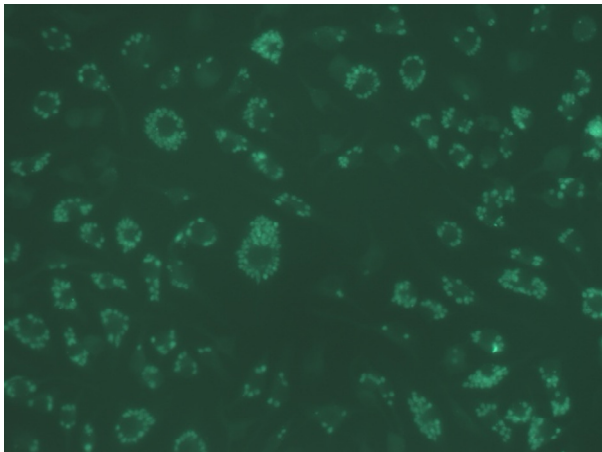

D

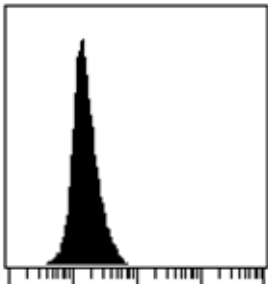

E

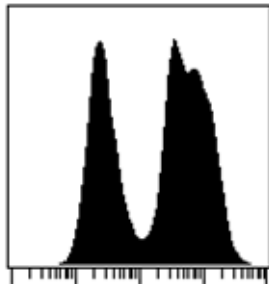

CFSE

CFSE

Supplement: Figure S3 — Infection of BMM with L. mexicana. (A) Uninfected BMMs stained with Diff-Quik. (B) BMMs infected with L. mexicana at a MOI of 10 and stained with Diff-Quik 24 h post-infection. (C) BMMs infected with CFSE labelled L. mexicana at a MOI of 10, visualized by fluorescent microscopy. (D) Flow cytometry analysis on uninfected BMMs (D) and BMMs infected with CFSE-labelled L. mexicana at a MOI of 10 (E). (0.49 MB PDF) [file pntd.0000648.s006.pdf]

Supplementary Figure 4

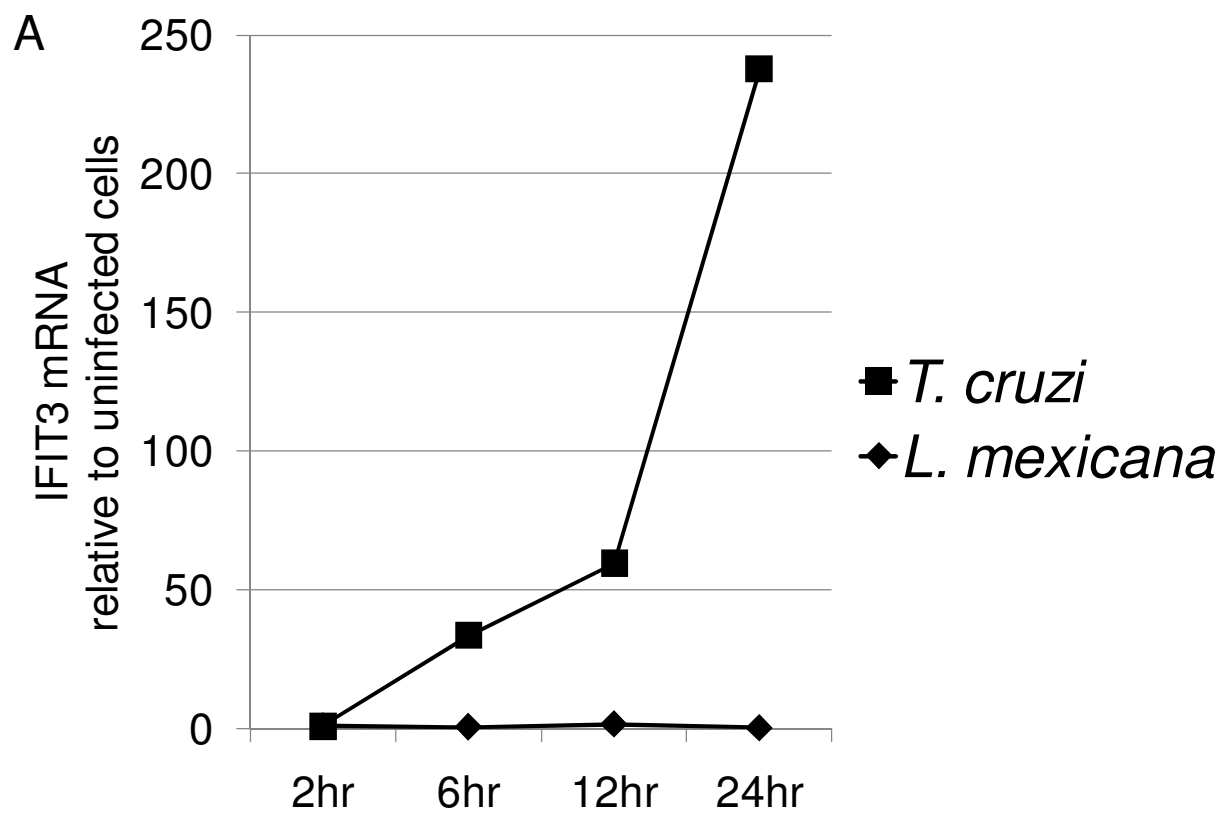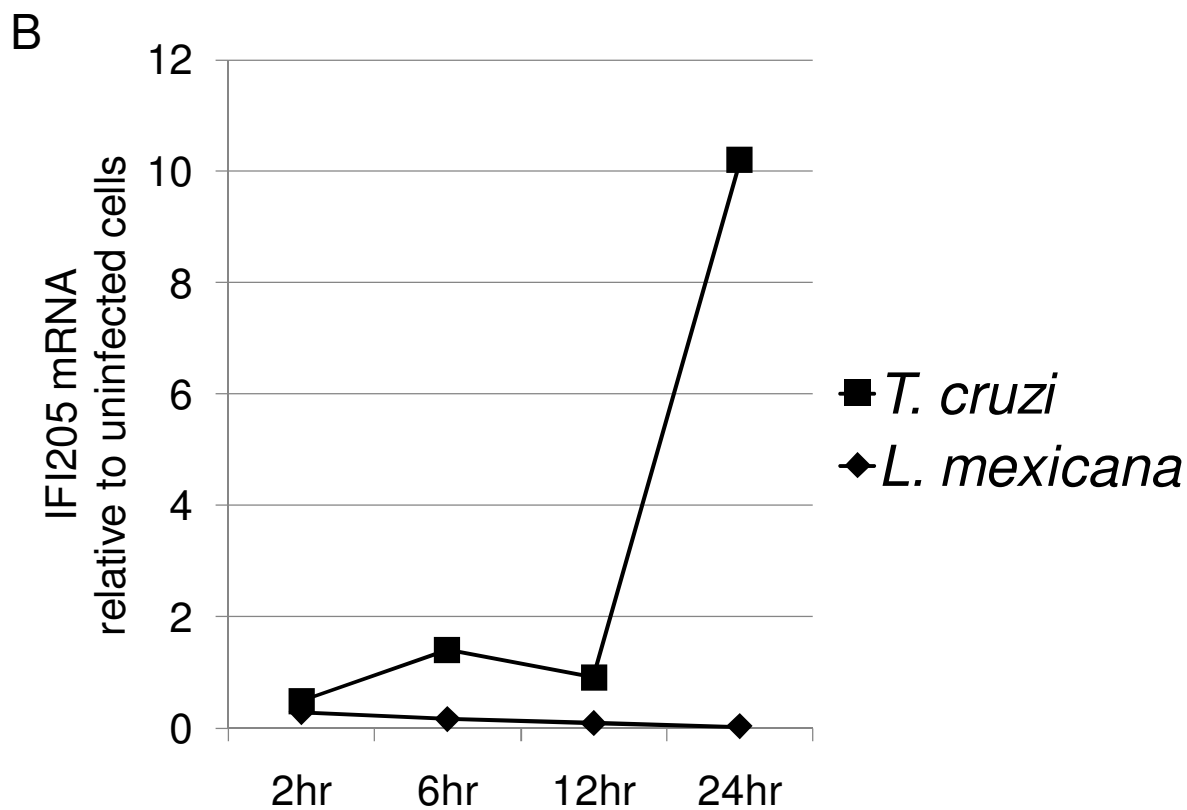

Supplement: Figure S4 — Induction of interferon-stimulated genes by T. cruzi. Quantitative real-time PCR analysis on cDNA from cells infected with T. cruzi or L. mexicana and from uninfected cells using primers directed against the interferon-stimulated genes IFIT3 (A) and IFI205 (B). (0.01 MB PDF) [file pntd.0000648.s007.pdf]

Supplementary Figure 5

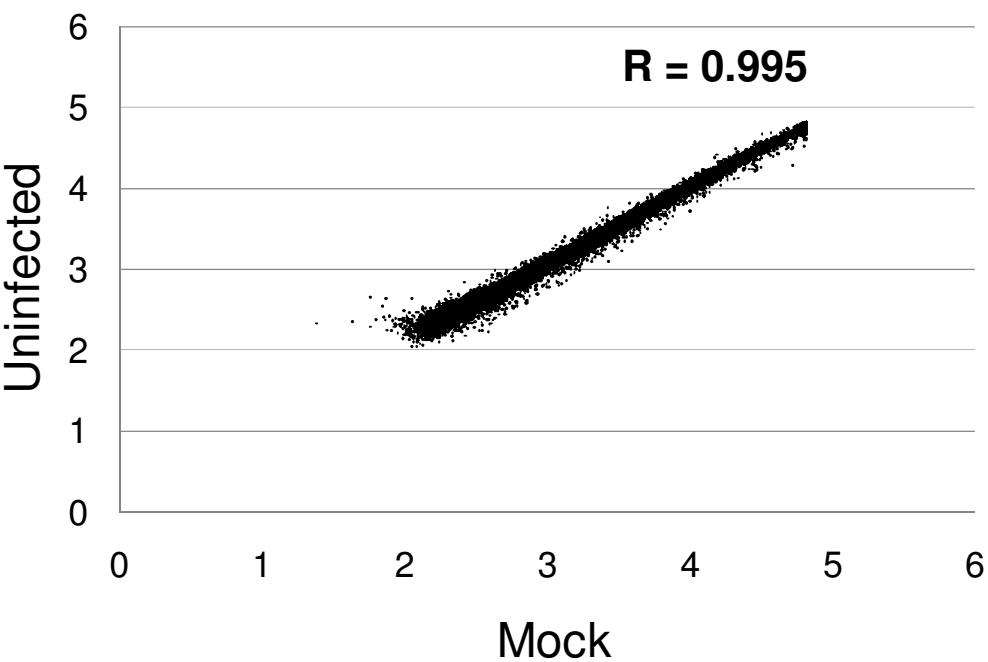

Supplement: Figure S5 — Comparative analysis of uninfected versus T. cruzi mock-infected BMMs. RNA from uninfected BMMs and BMMs treated with supernatant from uninfected BESM cells for 24 h (mock-infected BMMs) were collected and hybridized post-amplification against each other (uninfected BMM RNA labelled with Cy3 and mock-infected BMM RNA labelled with Cy5) on a MEEBO oligonucleotide array. The scatter plot shows the resulting median fluorescence intensities plotted on the X and Y axis for fresh and frozen macrophages. The correlation coefficient (R) is shown. (0.88 MB PDF) [file pntd.0000648.s008.pdf]

# Supplementary Figure 6

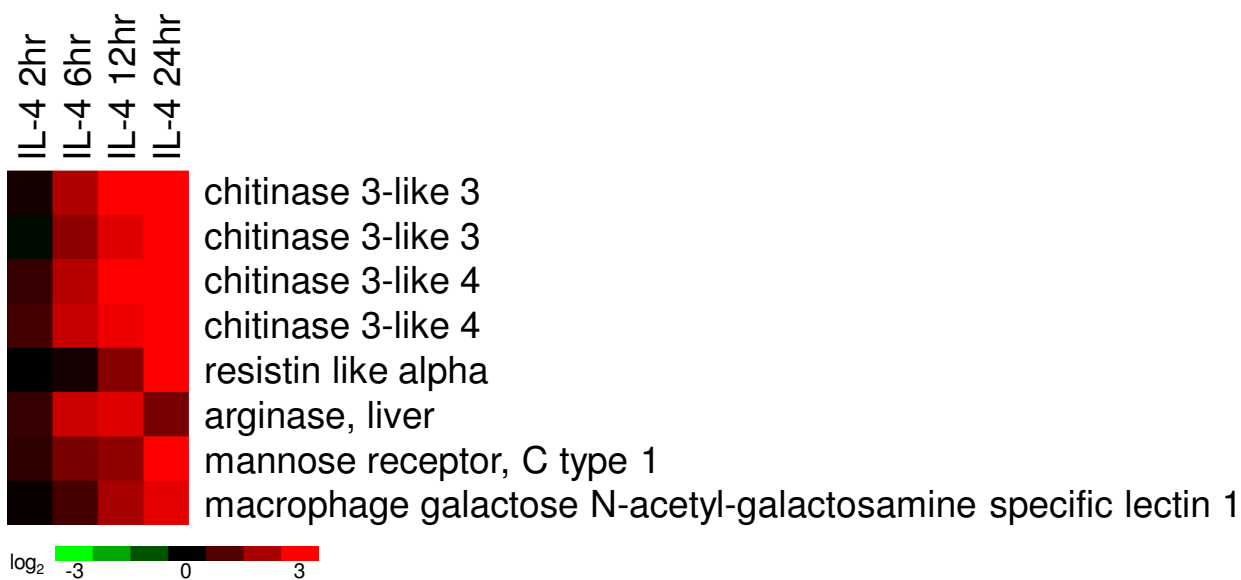

Supplement: Figure S6 — Induction of alternative macrophage activation markers by IL-4 stimulated BMMs. Heatmap showing the expression of genes that are known to be induced by IL-4 in alternatively activated macrophages, extracted from our IL-4 time course data. Black indicates unchanged level of expression relative to time 0 h, and red indicates upregulated levels expression. (0.01 MB PDF) [file pntd.0000648.s009.pdf]

# Supplementary Figure 7

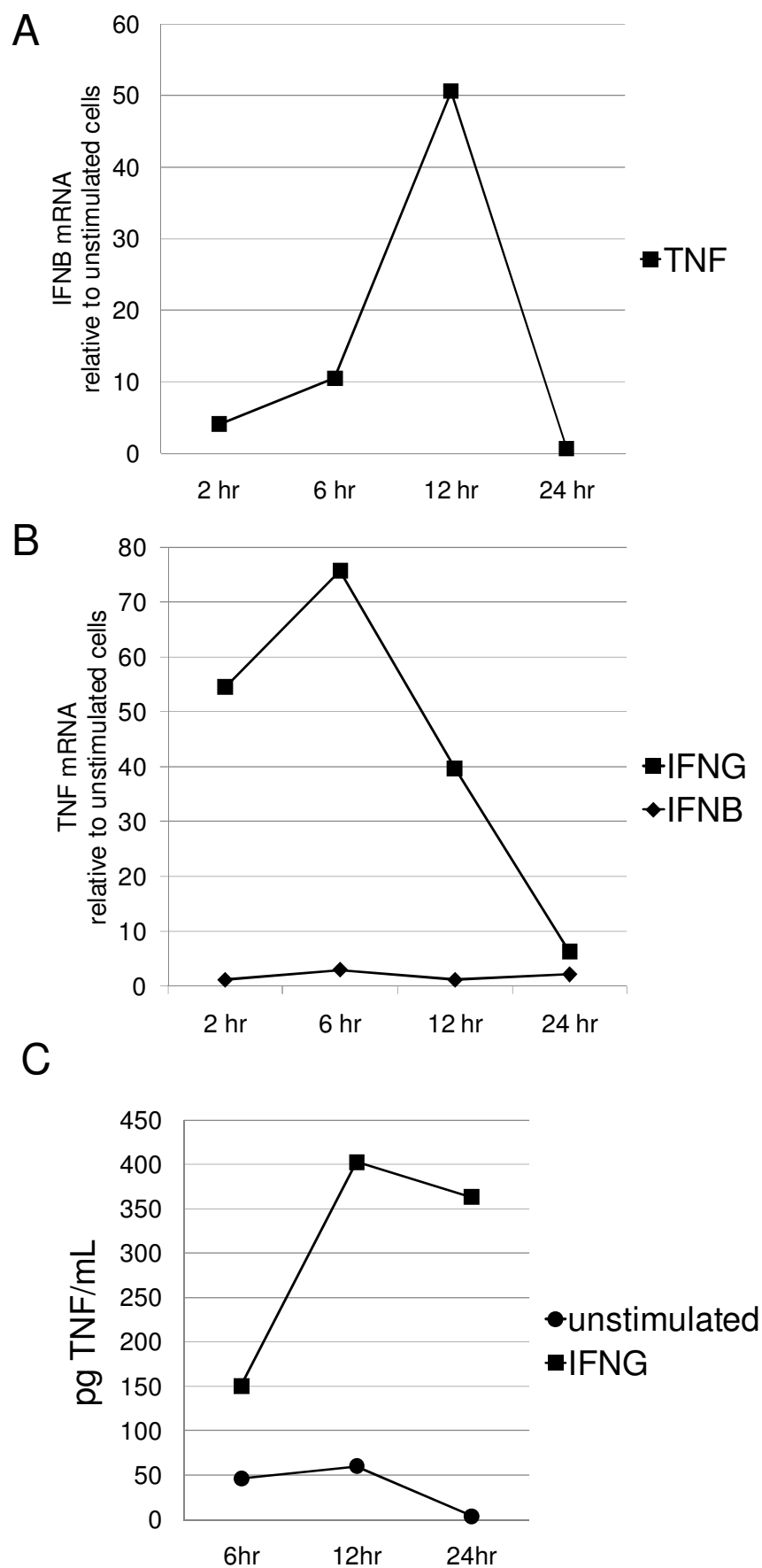

Supplement: Figure S7 — Cross induction of classical activation cytokines. (A) Quantitative real-time PCR analysis of Ifnb expression in cells stimulated with recombinant TNF and on unstimulated cells. TNF induced production of Ifnb transcript by 6 h post-stimulation. (B) Quantitative real-time PCR analysis of Tnf expression in cells stimulated with recombinant IFNG and IFNB and on unstimulated cells. TNF induced production of Ifnb transcript by 6 h post-stimulation. IFNG induced expression of Tnf transcript by 2 h post-stimulation, but IFNB does not. (C) TNF protein secretion into the supernatant of IFNG-stimulated BMMs 6 h post treatment was measured by cytometric bead analysis (BD). (0.01 MB PDF) [file pntd.0000648.s010.pdf]

Supplementary Figure 8

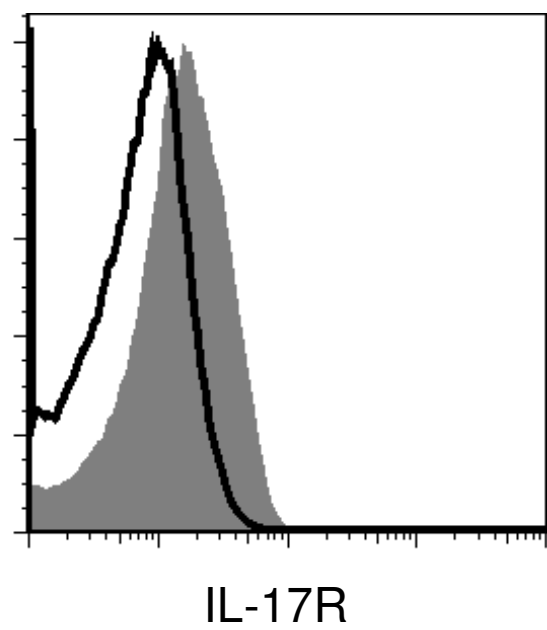

Supplement: Figure S8 — IL-17 receptor expression on bone marrow-derived macrophages Cell surface antigen staining was performed on BMMs using PE-conjugated IL-17R antibody. The histogram shows cells stained with IL-17R (filled) and cells stained with IgG2a isotype control (unfilled). (0.01 MB PDF) [file pntd.0000648.s011.pdf]

Supplementary Figure 9

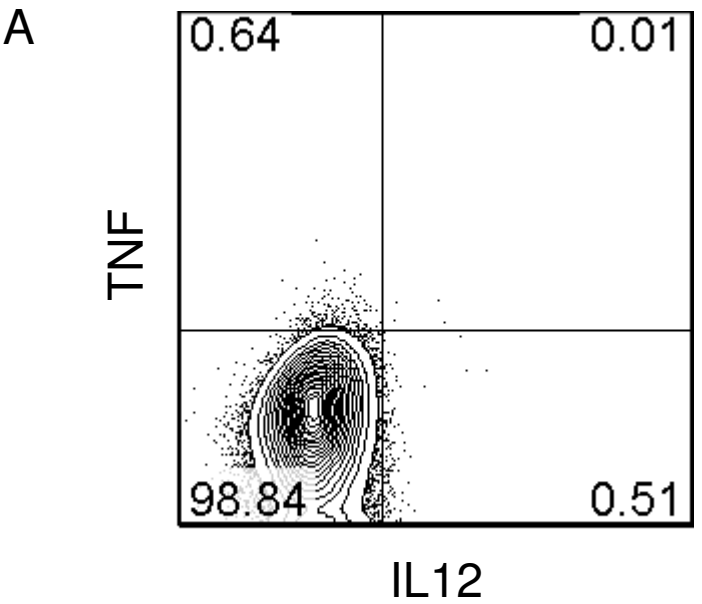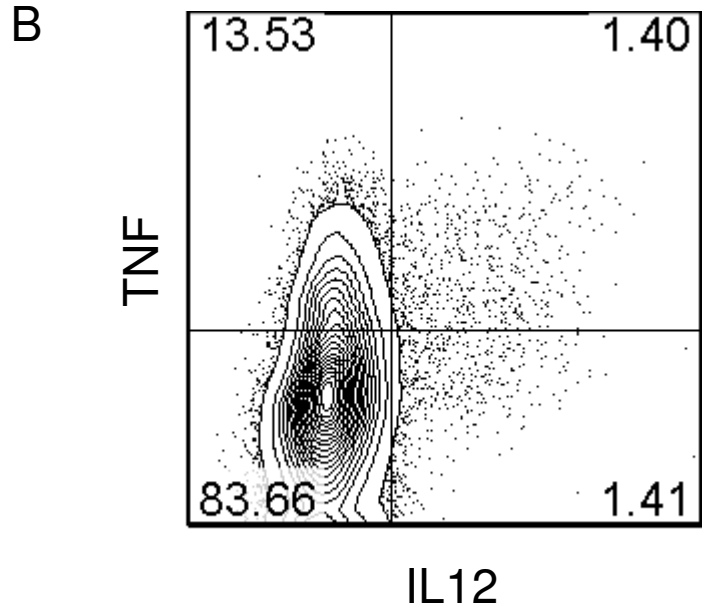

Supplement: Figure S9 — Induction of TNF by BMMs activated with LPS. Intracellular cytokine staining analysis of TNF production in unstimulated BMMs (A) and BMMs stimulated with 100 ng/uL of LPS for 4 h (B). (0.02 MB PDF) [file pntd.0000648.s012.pdf]

Supplementary Figure 10

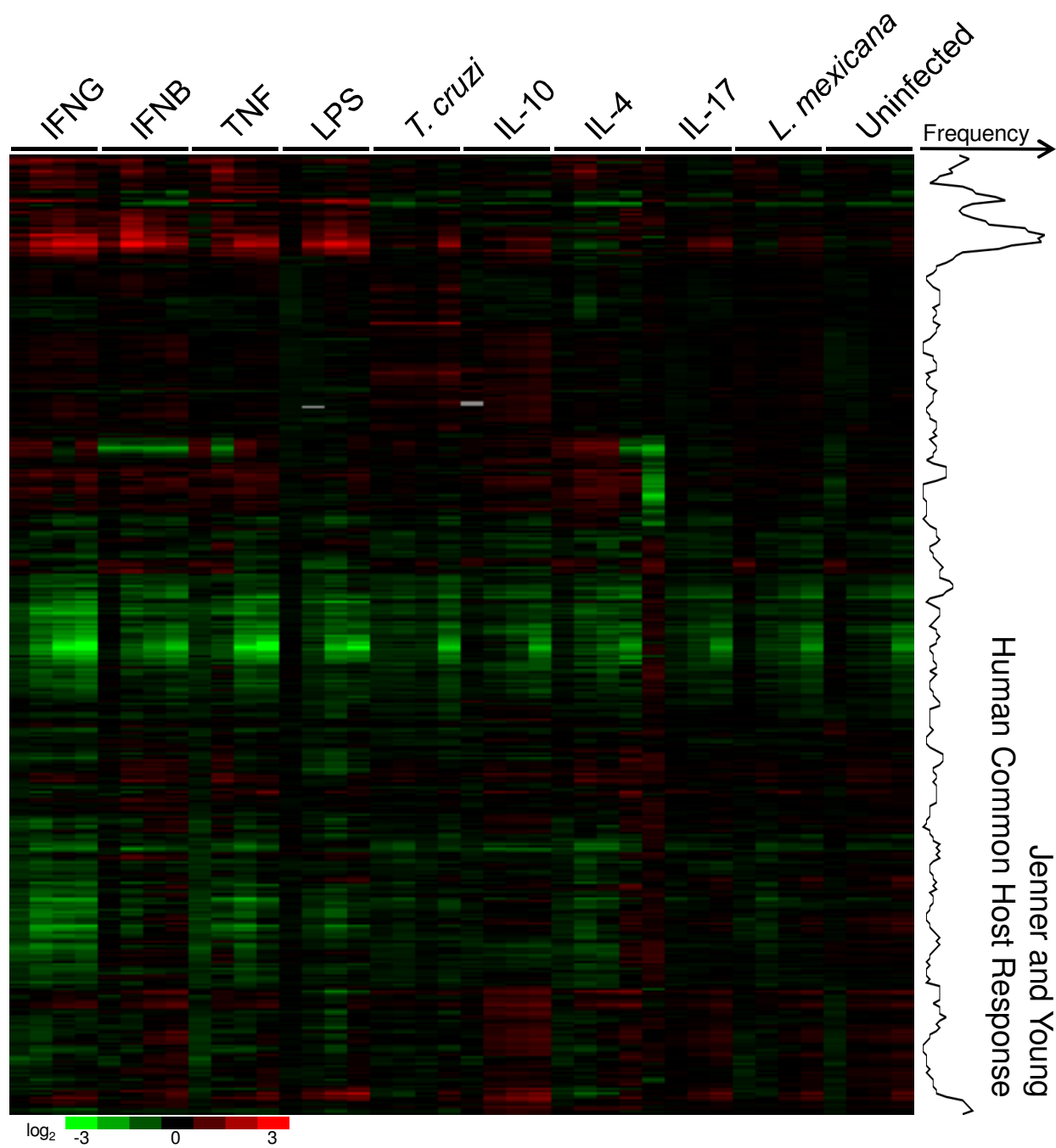

Supplement: Figure S10 — Relating mouse macrophage responses to the human “common host response” Heat map showing significantly altered genes as determined by multiclass SAM analysis for all cytokine and pathogen arrays (n = 5414). The graph on the right side represents the frequency that genes in the heat map appear in the human common host response set determined by Jenner and Young [47]. (0.06 MB PDF) [file pntd.0000648.s013.pdf]
