# Supplementary material for: Delineation of Diverse Macrophage Activation Programs in Response to Intracellular Parasites and Cytokines
Source: PLoS Negl Trop Dis. 2010 Mar 30;4(3):e648. doi: 10.1371/journal.pntd.0000648 (PMC2846935; doi:10.1371/journal.pntd.0000648)
Supplement: Methods S1 — Methods for Supporting Information materials. (0.06 MB DOC) [file pntd.0000648.s014.doc]

**Supplementary Methods**

**Cryopreserved bone marrow macrophages**

Newly derived BMMs and previously frozen BMMs were plated overnight in BMM media. RNA was collected from the fresh and previously frozen BMMs and prepped for microarray analysis. RNA from fresh BMMs was labelled with Cy5 and hybridized directly against RNA from previously frozen BMMs (labelled with Cy3). The background subtracted median fluorescence intensities of each color channel was plotted against one another, and linear regression analysis was performed. The correlation coefficient was found to be 0.986 (Supplementary Figure 1), suggesting that cryopreservation does not affect the transcriptional signature of BMMs.

**Mock infection of *T. cruzi***

BMMs were treated with supernatant from uninfected BESM cells in parallel with BMMs treated with supernatant from BESM cells infected with *T. cruzi*. RNA was collected from mock-infected BMMs 24 h post-treatment. RNA from mock-infected BMMs were processed for microarray analysis, labeled with Cy5 dye, and hybridized against RNA from uninfected BMMs (labeled with Cy3 dye). The median fluorescence intensities of mock-infected and uninfected cells were plotted against one another, and linear regression analysis was performed. The correlation coefficient was found to be 0.995 (Supplementary Figure 5), suggesting that supernatant from uninfected BESM cells did not affect the transcriptional signature of BMMs.

***Leishmania* and *Trypanosoma* meta-analysis**

Raw Affymetrix CEL files were obtained from the following repositories: studies by Fortea, Chessler, Costales, Shighara from GEO (http://www.ncbi.nlm.nih.gov/geo/), studies by Ettinger and Fisher from ArrayExpress (http://www.ebi.ac.uk/microarray-as/ae/), and the study by Chaussabel from GPX-MEA (http://gpxmea.gti.ed.ac.uk/). Processed Affymetrix data was obtained for the study by Gregory from ArrayExpress. For the study by Rodriguez, processed data was obtained directly from the author. For the spotted cDNA study by Vaena de Avalos, filtered normalized ratio of means for all spots on all arrays, was obtained from SMD (http://smd.stanford.edu/) using the following filters: (SF >=0) and (regression correlation > .6) and ((% pixels ch1 > BG ch1 + 1SD > 70) or (% pixels ch2 > BG ch2 + 1SD > 70)) .

For the studies with Affymetrix CEL files (Chaussabel, Fortea, Chessler, Costales, Shighara, Ettinger, and Fisher) normalization was done by using the justRMA function in the affy library in R (http://www.bioconductor.org/packages/release/Software.html). For the studies with the processed Affymetrix data (Gregory and Rodriguez), we used the authors' MAS5 normalized data. Expression values < 50, were floored at 50.

For the studes with Affymetrix CEL files, the function detection.p.val from the R library simpleaffy (http://www.bioconductor.org/packages/release/Software.html) was used to obtain probe set present, marginal, or absent calls in each array. Probe sets absent in all arrays were removed. For the studies with the processed Affymetrix data, we used the authors' present, marginal, or absent calls to filter out probe sets that were absent in all arrays.

Human HGNC gene identifiers along with all the corresponding fields were obtained (http://www.genenames.org/data/gdlw_index.html). Probe sets for the Affymetrix arrays were mapped to the HGNC gene identifiers using the human gene symbols provided by the corresponding Affymetrix gene list (http://www.affymetrix.com/). For the Vaena de Avalos study, the human gene symbols provided for the data from SMD were mapped directly to the HGNC gene identifiers.

For the MEEBO arrays, oligos were mapped to mouse gene identifiers from MGI using the mouse to human gene orthology table obtained from the MGI website (http://www.informatics.jax.org/).

**Jenner & Young ortholog mapping**

Mouse genes were organized by hierarchical clustering using a Pearson correlation distance metric. To assess the degree of similarity between the mouse and human responses to pathogens, mouse orthologs for the human “common host response” expression signature identified by Jenner and Young [33] were mapped to the MEEBO probe set using Mouse Genome Informatics and NCBI ortholog assignments. 474 of the 511 human common host response genes were mapped to MEEBO probes. Of these, 239 human common host response genes mapped to the probes in the mouse dataset (*i.e.* those which passed all filtering criteria). The mapped human genes were organized in the same order in which the mouse genes appear in the cluster, and the frequency of appearance was tallied in blocks of 50 genes. Frequencies were smoothed using a moving window average of 5 and plotted along the gene axis of the cluster heatmap in order to demonstrate the location of the human response genes relative to the responding genes in the mouse dataset.

**Quantitative real-time PCR**

Two micrograms of RNA from each sample was reverse transcribed in a 20 μL reaction. One microliter of the resulting cDNA was used in quantitative real-time PCR reactions with SYBR green labelling. All values were normalized to GAPDH values. The following primers were used for qRT-PCR: *gapdh*-F – 5’-AACTTTGGCATTGTGGAAGG, *gapdh*-R – 5’- ACACATTGGGGGTAGGAACA, *ifnb*-F – 5’-CTGGAGCAGCTGAATGGAAAG, *ifnb*-R – 5’-CTTGAAGTCCGCCCTGTAGGT, *tnfa*-F – 5’-GCACCACCATCAAGGACTCAA, *tnfa*-R – 5’-TCGAGGCTCCAGTGAATTCG, *ifit3*-F – 5’- CTGAACTGCTCAGCCCACAC, *ifit3*-R – 5’- TGGACATACTTCCTTCCCTGA, *ifi205*-F – 5’- TCCACAACCCAGGAAGAGAC, *ifi205*-R – 5’- GAAGCCGAAGATGAGACCTG.

**Flow cytometry**

Cell surface marker analysis was performed to confirm the purity of bone marrow-derived macrophages. BMMs were thawed and plated overnight in BMM media. The following day the cells were washed twice with PBS, and taken off the plate using a cell scraper. The cells were then treated with Fc Block (BD Biosciences), incubated for 15 m, and then stained with antibodies. Six antibodies were used in the analysis: FITC-CD11c (eBiosciences), PE-CD11b (eBiosciences), PeCy5-F4/80 (eBiosciences), APC-GR1 (BD Biosciences), APC-CD19 (BD Biosciences), and APC-CD3 (BD Biosciences). After a 15 m incubation in the dark, cells were washed and resuspended in 500 uL of PBS + 10% FBS for flow cytometry analysis. The analysis was performed using a BD FACScalibur system with four-color florescence capability.

Cell surface markers were chosen to represent a range of immune cells. CD11c is a marker for dendritic cells [34,35]. CD11b is a marker for macrophages [36,37,38]. F4/80 is considered the best marker for identifying mature macrophages [39,40]. GR1 is a marker for bone marrow granulocytes and peripheral neutrophils [41,42]. CD19 is a marker for B cells [43,44]. CD3 is a marker for T cells [45]. As our population of BMMs were not expected to be positive for GR1, CD19, and CD3, antibodies chosen for these markers were all conjugated to a single florescent marker (APC). We found that 99.4% of the BMMs were CD11b+ (Supplementary Figure 2a), and 93.8% were F4/80+ (Supplementary Figure 2b). The cells stained negative for CD11c, GR1, CD19, and CD3 (data not shown).

Flow cytometry analysis was performed to confirm infectivity of *L. mexicana* in bone marrow-derived macrophages. BMMs were thawed and plated overnight on 6-well plates. *L. mexicana* were labelled with 10uM CFSE (Invitrogen) for 5 m at 37°C, mixing every 2 m. After 5 m, an equal volume of FBS was added to the labelled parasites, and the tube was spun down and washed with 15 mL of DMEM + 10% FBS. Labelled parasites were then resuspended in DMEM + 0.5% FBS for infection. BMMs were infected at a MOI of 10 and harvested 6 h post-infection using a cell scraper. The cells were washed with PBS and fixed in PBS + 0.2% formaldehyde for flow cytometry analysis.

Cell surface antigen staining and flow cytometry analysis were used to determine IL-17 receptor expression on BMMs. BMMs were thawed and stained with PE-conjugated anti-mouse IL-17R (eBiosciences). IL-17R-stained BMMs were compared to BMMs stained with PE rat IgG2a isotype control (eBiosciences) (Supplementary Figure 8).

Flow cytometry was also used to measure TNF and IL-12 production by BMMs stimulated with LPS. BMMs were thawed and plated overnight on 6-well plates. The next day 1 uM Monensin (BD Biosciences) and 100 ng of LPS (Sigma) were added to the cells. Four hours later, intracellular cytokine staining was performed by harvesting the cells using a cell lifter and resuspending the cells in 250 uL Cytofix/Cytoperm (BD Biosciences). The cells were incubated for 10 m at 4°C. They were then washed with Perm/Wash solution (BD Biosciences) and stained with PE-conjugated IL-12 (BD Biosciences) and APC-conjugated TNF (BD Biosciences). After incubating for 15 m in the dark, the cells were washed again Perm/wash and resuspended in 200 uL PBS + 200 uL 0.4% Formaldehyde.

**Cytometric bead analysis**

BMMs were plated in 96-well plates at a density of 10^5^ cells/well and stimulated with 100 ng/mL IFNG (R&D Systems). Supernatants were collected from wells at 2 h, 6 h, 12 h, and 24 h post-infection. Cytokine stimulation was performed in triplicate (3 wells per time point), and the supernatants from the 3 wells were pooled for the analysis. Cell supernatants were analyzed using the Cytometric bead array mouse inflammation kit (BD Biosciences) by following the manufacturer’s instructions. Data was collected using a BD Facscalibur.

**Cell staining**

BMMs infected with *L. mexicana* along with uninfected BMMs were stained at 24 h post-infection by Diff-Quik and mounted using Prolong with Dapi (Invitrogen).
